# Influence of Simvastatin and Pravastatin on the Biophysical Properties of Model Lipid Bilayers and Plasma Membranes of Live Cells

**DOI:** 10.1021/acsbiomaterials.4c00911

**Published:** 2024-08-24

**Authors:** Artu̅ras Polita, Ru̅ta Bagdonaitė, Arun Prabha Shivabalan, Gintaras Valinčius

**Affiliations:** Department of Biospectroscopy and bioelectrochemistry, Institute of Biochemistry, Life Sciences Center, Vilnius University, Saulėtekio av. 7, Vilnius LT-10257, Lithuania

**Keywords:** simvastatin, pravastatin, model lipid bilayers, BODIPY, viscosity, lipid order, FLIM

## Abstract

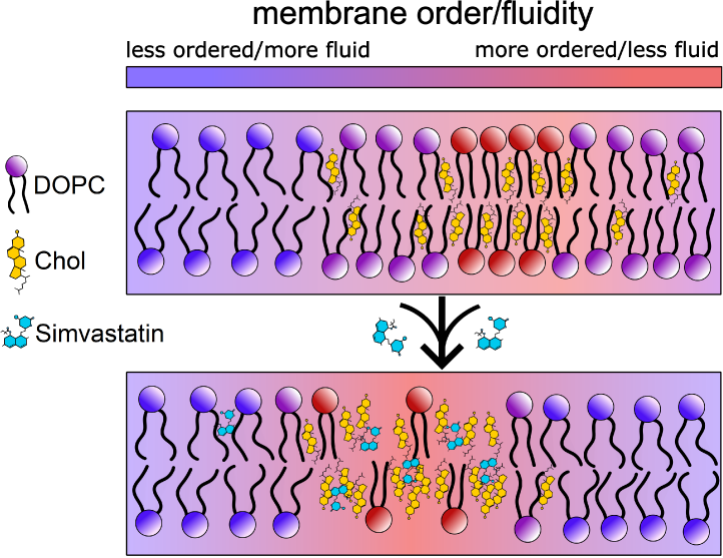

Statins are among the most widely used drugs for the inhibition of cholesterol biosynthesis, prevention of cardiovascular diseases, and treatment of hypercholesterolemia. Additionally, statins also exhibit cholesterol-independent benefits in various diseases, including neuroprotective properties in Alzheimer’s disease, anti-inflammatory effects in coronary artery disease, and antiproliferative activities in cancer, which likely result from the statins’ interaction and alteration of lipid bilayers. However, the membrane-modulatory effects of statins and the mechanisms by which statins alter lipid bilayers remain poorly understood. In this work, we explore the membrane-modulating effects of statins on model lipid bilayers and live cells. Through the use of fluorescence lifetime imaging microscopy (FLIM) combined with viscosity-sensitive environmental probes, we demonstrate that hydrophobic, but not hydrophilic, statins are capable of changing the microviscosity and lipid order in model and live cell membranes. Furthermore, we show that hydrophobic simvastatin is capable of forming nanoscale cholesterol-rich domains and homogenizing the cholesterol concentrations in lipid bilayers. Our results provide a mechanistic framework for understanding the bimodal effects of simvastatin on the lipid order and the lateral organization of cholesterol in lipid bilayers. Finally, we demonstrate that simvastatin temporarily decreases the microviscosity of live cell plasma membranes, making them more permeable and increasing the level of intracellular chemotherapeutic drug accumulation.

## 1 Introduction

**Figure 1:**
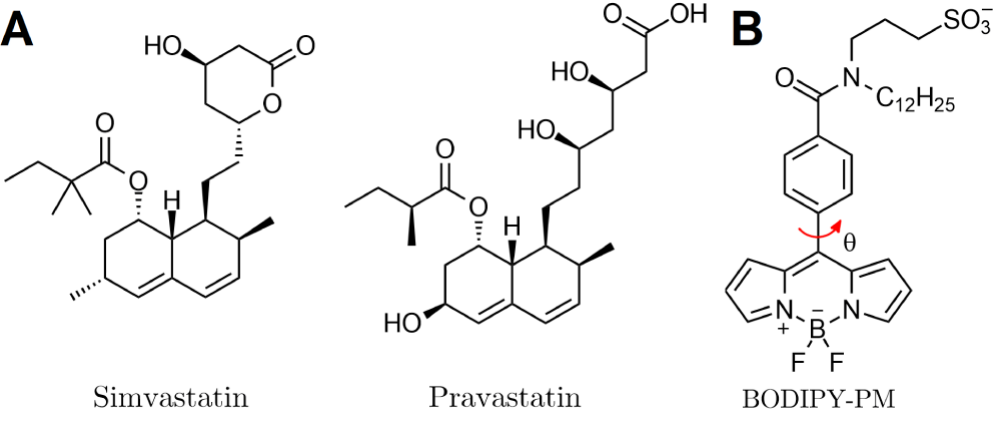
(A) Structures of statins: lipophilic simvastatin and hydrophilic pravastatin. (B) Structure of molecular rotor BODIPY-PM. The red arrow indicates intramolecular rotation, which causes BODIPY-PM to display sensitivity to viscosity.

Statins are one of the most commonly used drugs for treating hypercholesterolemia and reducing the risk of cardiovascular disease. In addition to the inhibition of HMG-CoA reductase, the rate-limiting step in cholesterol biosynthesis,^1^ increasing evidence suggests that statins also interact with lipid membranes^2,3^ and affect their physical properties – increase the heterogeneity and fluidity of lipid bilayers,^4−6^ influence the activity of cholesterol-dependent toxins,^7^ and increase the order of lipids in model membranes.^8^ Lipophilicity plays a significant role in statins’ ability to interact with lipid membranes and manifest their biophysical effects.^9^ Simvastatin (Figure 1A), being approximately 100 times more lipophilic than pravastatin,^10^ has been shown to have a significantly greater lipid ordering effect compared to pravastatin.^8,11^ Additionally, hydrophobic, but not hydrophilic, statins have been shown to reduce the membrane binding ability of cholesterol-dependent cytolysins.^7^ It has been suggested that statin-induced membrane fluidity changes may play a key role in statin-associated myopathy,^12^ where patients experience muscle pain and weakness due to the accumulation of lipophilic statins in skeletal myocytes.^13−15^ In addition, statins, and especially simvastatin, have received significant attention due to their ability to suppress the growth, proliferation, and migration of cancer cells^16,17^ by inducing apoptosis^18,19^ and nuclear fragmentation.^20^ Although numerous studies explore cholesterol-independent benefits of statins in various diseases, such as anti-inflammatory effects in coronary artery disease,^21^ immunomodulatory applications in rheumatoid arthritis,^22^ antiproliferative characteristics in cancer,^16−19^ and neuroprotective properties in Alzheimer’s disease,^23^ the effects of statins on cellular membranes, which may influence the said properties, remain poorly investigated. A detailed understanding of how statins affect the lipid bilayers could be of use in the development of drugs capable of altering the organization of plasma membranes^24^ and provide new treatments for a wide range of diseases, including multidrug-resistant cancers, neurodegenerative diseases, stroke, and diabetes.^25^ Finally, an in-depth knowledge of the impact of statins on the plasma membrane organization may be useful for elucidating the neuroprotective benefits of statins in Alzheimer’s disease^26^ or the antiproliferative properties of statins in cancer.^27^

Microviscosity measurements are one of the most convenient ways to observe the modulatory effects of small molecules, such as statins, on the lipid bilayers.^28^ The term microviscosity here refers to the lipid packaging and molecular mobility of the probe in a local environment. The microviscosity values of lipid bilayers are heavily influenced by the efficiency of lipid packaging and the order of lipids: for example, cholesterol-rich bilayers form highly viscous liquid-ordered phases, whereas highly unsaturated lipids produce nonviscous disordered phases.^29,30^ Viscosity-sensitive dyes, known as molecular rotors, are able to quantify the microviscosity variations caused by cholesterol differences or phase separations in lipid bilayers.^31−33^ In the excited state, molecular rotors can undergo intramolecular rotation, resulting in a molecular rotor entering the dark state.^34^ Thus, in low viscosity, or noncrowded lipid bilayers, the intramolecular rotation of the rotor is not hindered, and the nonradiative decay dominates, resulting in a decrease of the fluorescence quantum yield and lifetime.^35^ When paired with fluorescence lifetime imaging microscopy (FLIM), molecular rotors are able to produce spatial microviscosity maps of lipid structures and reveal dynamical changes in the membranes.^36−38^

In this work, we investigate the structural changes induced by hydrophobic simvastatin and hydrophilic pravastatin in model tethered bilayer lipid membranes (tBLMs) and plasma membranes of live cancer and noncancer cells. With the use of viscosity-sensitive and plasma membrane-specific molecular rotor BODIPY-PM (Figure 1B),^39^ we show that simvastatin induces the formation of highly viscous circular nanoscale domains in cholesterol-rich tBLMs. To the best of our knowledge, none of the studies so far have reported microscopic evidence for the formation of nanoscale domains. Our results demonstrate that simvastatin’s lipid ordering and disordering effects are simvastatin concentration-dependent. At low concentrations, by transferring cholesterol from the lipid bilayer into the circular nanoscale domains, simvastatin decreases the order of the lipids. At high concentrations, simvastatin increases the order of the lipids by partitioning into the domain-unaffected areas of the lipid bilayer. Furthermore, we show that prior to circular domain formation, simvastatin is capable of homogenizing the cholesterol concentration throughout the lipid bilayer. To the best of our knowledge, none of the studies so far have reported microscopic evidence of statin-induced nanoscale domain formation or cholesterol homogenization effects in lipid bilayers. In contrast, we demonstrate that pravastatin has no effect on the microviscosities of tBLMs or the plasma membranes of live cells. Our findings provide a mechanistic framework for understanding the bimodal effects of hydrophobic statins and the organizational changes they induce in model lipid bilayers. Finally, we demonstrate that simvastatin uniformly reduces the plasma membrane microviscosities of live lung cancer (A549) and immortalized human kidney (HEK 293T) cells. Simvastatin-decreased plasma membrane microviscosities result in an increased intracellular level of the accumulation of the chemotherapeutic drug doxorubicin in A549 cells. Furthermore, the effects of simvastatin on live cell plasma membranes are temporal, and in 4 h, the cells recover their initial plasma membrane microviscosity values.

## 2 Results and Discussion

### 2.1 Imaging of Statin-Induced Microviscosity Changes in tBLMs

**Figure 2:**
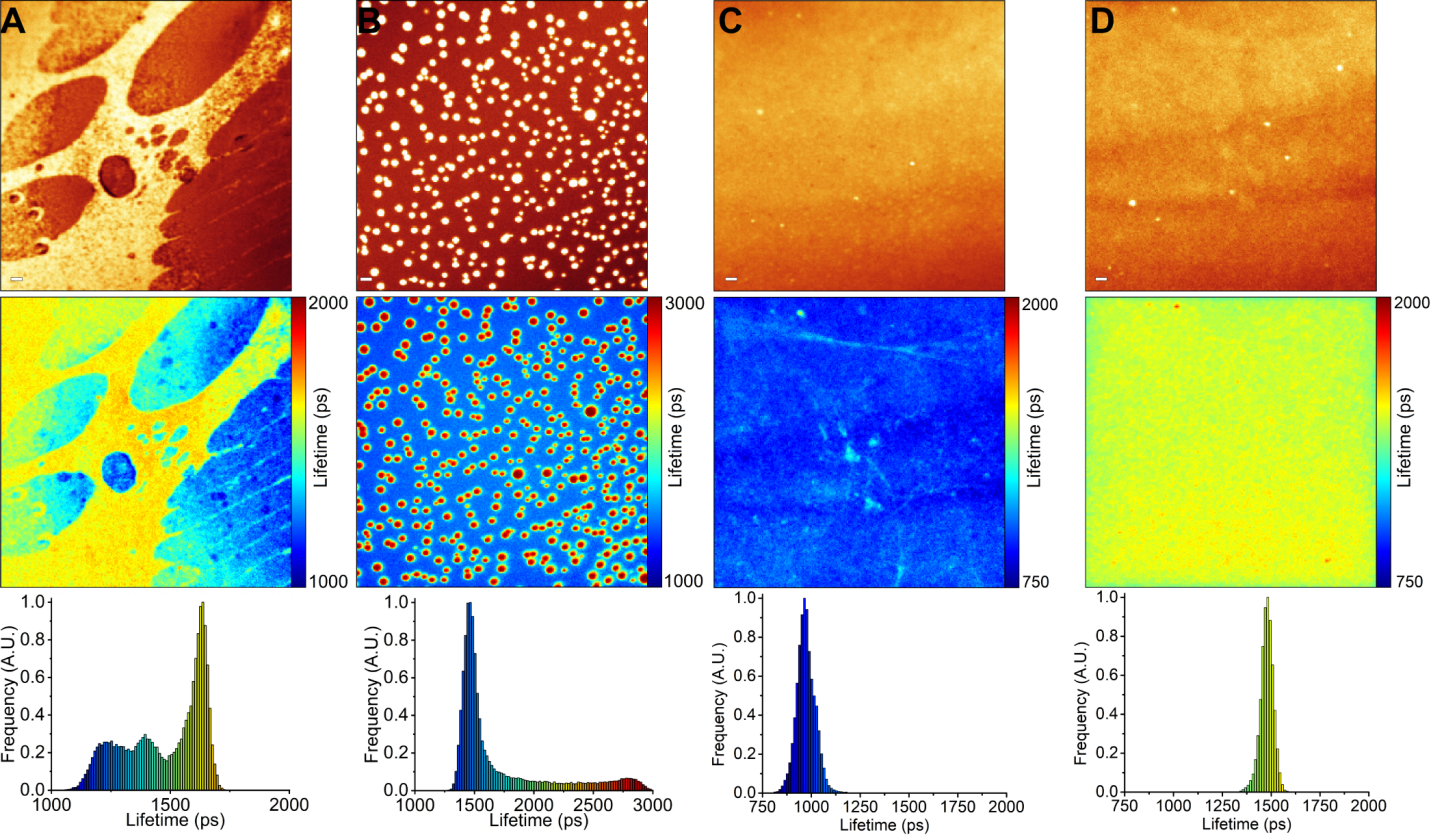
FLIM of BODIPY-PM in DOPC/Chol 60/40 and DOPC tBLMs. (A) DOPC/Chol tBLM before simvastatin addition. (B) DOPC/Chol tBLM 5 min after addition of simvastatin. (C) DOPC tBLM before simvastatin addition. (D) DOPC tBLM 5 min after addition of simvastatin. The top panel shows images of the fluorescence intensity. FLIM images are shown in the middle panel. The corresponding lifetime histograms are shown at the bottom panel. Scale bars are 1 μm.

To investigate the effects of statins on lipid bilayers, we performed FLIM measurements on DOPC/Chol 60/40 and pure DOPC tBLMs stained with BODIPY-PM (Figure 2). We note that tBLMs stochastically form heterogeneous lipid bilayers with irregularly shaped cholesterol-rich areas that feature greater microviscosities compared to their surroundings (Figure 2A). The addition of simvastatin (10 μM) to DOPC/Chol 60/40 tBLMs resulted in the formation of circular, highly viscous domains ranging in size from 300 to 700 nm (Figure 2A,B). The intensity-weighted fluorescence lifetimes of BODIPY-PM in the simvastatin-induced domains vary from 2500 to 3000 ps, corresponding to the microviscosity values of 170–250 cP in methanol–glycerol calibration mixtures.^39^ High intensity-weighted fluorescence lifetimes of BODIPY-PM indicate that the circular domains are both fluid and highly ordered, since large unilamellar vesicles produced from the fluid liquid-ordered lipid phase, as well as eukaryotic live cell membranes, display fluorescence lifetimes of about 3300 and 4500 ps, respectively.^39^ Substantial microviscosity differences between viscous domains (170–250 cP) and the remainder of the lipid bilayer (about 70 cP in cholesterol-poor regions and 90 cP in cholesterol-rich regions) demonstrate that simvastatin addition induces phase separation in the lipid bilayer.

We also note that the fluorescence lifetime gradient observed on the exterior of the domains is caused by the diffraction limit of fluorescence microscopy and is a consequence of the combined fluorescence lifetimes of BODIPY-PM from both the domain and adjacent areas at the phase separation boundaries (Figure 2B). If boundary regions of the domains with fluorescence lifetime gradients are omitted, the microviscosities of single domains appear to be uniform, indicating that only one phase exists within the domain. Simultaneously with the formation of circular domains, the intensity-weighted fluorescence lifetimes of BODIPY-PM in cholesterol-rich areas decreased from about 1650 ps (Figure 2A, yellow areas in FLIM) to about 1400 ps (Figure 2B, blue areas) in regions where no domain formation occurred. In contrast, the cholesterol-poor areas (Figure 2A, blue), with intensity-weighted fluorescence lifetimes of about 1250 ps, became more ordered following simvastatin addition and displayed fluorescence lifetimes of about 1400 ps (Figure 2B). Given that cholesterol is the primary component responsible for ordering lipids and producing greater microviscosities in the binary DOPC/Chol system,^40^ we initially hypothesized that simvastatin may redistribute and homogenize cholesterol concentrations in the lipid bilayer. Furthermore, the circular viscous nanodomains form in both cholesterol-rich and cholesterol-poor areas of the bilayer, regardless of the homogeneity, implying either that the initial state of the bilayer is irrelevant for circular domain formation or that simvastatin induces cholesterol transfer and equalizes the cholesterol concentration throughout the bilayer prior to circular domain formation (Figure 2A,B). To test whether the initial homogeneity of the bilayer impacts the number, size, and microviscosity of simvastatin-induced domains, we performed analogous FLIM measurements in highly homogeneous areas of DOPC/Chol 60/40 tBLMs (Figure S1). The addition of simvastatin produced no discernible effects on the number, size, or microviscosity of circular domains in homogeneous regions of DOPC/Chol tBLMs compared to heterogeneous areas (Figure S1). Interestingly, all viscous domains following simvastatin addition appear to be circularly shaped rather than having an irregular form (Figures 2B and S1). We believe that the domains assume a circular shape to minimize the line tension at the phase-separation boundaries, implying that the shape of viscous domains is determined by the surface tension rather than by molecular interactions within the domain.

In contrast to DOPC/Chol 60/40 tBLMs, the addition of simvastatin to pure DOPC tBLMs did not result in the formation of circular viscous nanoscale domains (Figure 2C,D). Instead, the intensity-weighted fluorescence lifetimes of BODIPY-PM increased from about 900 to 1450 ps following simvastatin addition (Figure 2D). Thus, simvastatin’s bilayer-modulatory effects are cholesterol-dependent, and in the absence of cholesterol, simvastatin uniformly increases the order of lipid in the bilayer, whereas the presence of cholesterol leads to circular domain formation. The increase in microviscosity and lipid order of DOPC tBLMs after simvastatin addition suggests that simvastatin likely occupies cholesterol sites in the lipid bilayer and operates similarly to cholesterol, i.e., orders, and condenses disordered lipid phases. Finally, the addition of pravastatin (10 μM) to DOPC/Chol tBLMs did not result in any significant microviscosity changes or the appearance of viscous domains (Figure S3). This is an anticipated outcome since pravastatin is substantially more hydrophilic compared to simvastatin and has multiple hydroxyl groups (Figure 1A), which likely prevent the molecule from integrating deeply into the lipid bilayer.

**Figure 3:**
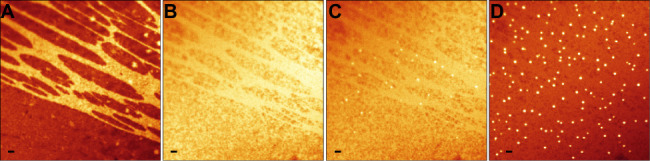
Fluorescence intensity images of BODIPY-PM in DOPC/Chol 60/40 tBLMs. (A) tBLM before simvastatin addition. (B) tBLM 1 min after simvastatin addition. (C) tBLM 3 min after simvastatin addition. (D) tBLM 5 min after simvastatin addition. Scale bars are 1 μm.

To further explore the mechanism of action of simvastatin on lipid bilayers, we decided to image the formation process of nanoscale domains in DOPC/Chol (60/40) tBLMs stained with BODIPY-PM (Figure 3). Although reliable quantitative microviscosity measurements are hardly possible in 10–30 s time frames in a rapidly changing environment, the quantum yield of BODIPY-PM and thus the fluorescence intensity increase in more ordered lipid environments,^39^ allowing for the detection of changes in lipid bilayer homogeneity. The addition of simvastatin initially resulted in the homogenization of the lipid bilayer, with cholesterol-rich (high-intensity) regions gradually dissipating in the first 3–4 min (Figure 3A,B). However, once the lipid bilayer became homogeneous, domain formation took place (Figure 3C,D). The quick homogenization of the lipid bilayer by simvastatin provides an explanation for the occurrence of analogous nanoscale domains in both heterogeneous and homogeneous areas of lipid bilayers: prior to domain formation, simvastatin equalizes the microviscosity and most likely the cholesterol concentration throughout the bilayer (Figure 3B). Thus, domain formation occurs from the same initial state (Figure 3C), regardless of whether the bilayer is homogeneous or heterogeneous. Consistently with the previously described experiments, the intensity-weighted fluorescence lifetimes of BODIPY-PM in circular-domain unaffected areas of the bilayer shifted from the initial 1600 to 1400 ps after simvastatin addition, indicating that simvastatin has decreased the lipid order of DOPC/Chol 60/40 tBLMs (Figure S4).

**Figure 4:**
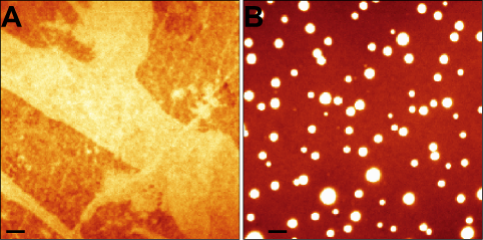
Fluorescence intensity images of Cy5 in DOPC/Chol tBLMs before simvastatin addition (A) and 5 min after simvastatin (10 μM) addition (B). Scale bars are 1 μm.

To verify that simvastatin truly homogenizes the cholesterol concentration across the bilayer and to investigate the composition of circular domains, we labeled cholesterol with the fluorescent cyanine dye Cy5 in DOPC/Chol 60/40 tBLMs. Analogous to the previous experiments, we imaged the fluorescence intensity of Cy5-Chol in heterogeneous areas of the lipid bilayer prior and after simvastatin (10 μM) addition. Consistent with our previous results, the addition of simvastatin resulted in the dissipation of stochastically formed cholesterol-rich areas and the formation of circular nanoscale domains (Figures 4 and S6). Furthermore, the fluorescence intensities of Cy5 in circular domains were about 20 times greater compared to the domain-free regions of the bilayer, revealing that cholesterol primarily localized in the circular domains (Figure 4B). The formation of cholesterol-rich circular domains and the gradual dissipation of cholesterol-rich areas both indicate that the addition of simvastatin induces cholesterol transfer, which initially manifests with the homogenization of the lipid bilayer and progresses to cholesterol-rich nanoscale domain formation. The decreased microviscosity and order of lipids in domain-free areas of the bilayer following simvastatin addition is, most likely, a result of cholesterol transfer from the bilayer to the nanoscale domains.

Finally, we investigated the influence of simvastatin concentration on the size and microviscosity of circular nanodomains: at 1 μM addition of simvastatin, the domain size ranges from 250 to 400 nm, with intensity-weighted fluorescence lifetimes of BODIPY-PM ranging from 2000 to 2500 ps, corresponding to the microviscosity values of 110–170 cP in methanol–glycerol calibration mixtures (Figure S7). We suspect that the observable size of the domains (about 300 nm) is limited by diffraction, and the actual dimensions could be significantly smaller. Similarly, if the observable size of the domains is limited by diffraction, the intensity-weighted fluorescence lifetimes of BODIPY-PM merely reflect the average microviscosity values between the domain and the domain-unaffected area of the lipid bilayer, implying that the actual microviscosities of the domains could be higher than 110–170 cP. In contrast to 10 μM simvastatin addition, 1 μM simvastatin addition significantly decreases the intensity-weighted fluorescence lifetimes of domain-unaffected areas of the bilayer to about 900 ps–a value closely similar to that of pure DOPC bilayers. At 20 μM addition of simvastatin, the average size of domains increases to 300–1200 nm and intensity-weighted fluorescence lifetimes of BODIPY-PM in domains increase to 3000–3500 ps, corresponding to the viscosity values of 250–320 cP in methanol–glycerol calibration mixtures (Figure S8). The domain-unaffected areas of the bilayer, however, display intensity-weighted fluorescence lifetimes of about 1500 ps. Finally, at 100 μM of simvastatin addition, the domains become micron-sized and the intensity-weighted fluorescence lifetimes of BODPY-PM increase to 4500–5000 ps, corresponding to the viscosity values of 680–880 cP in methanol–glycerol calibration mixtures (Figure S9). However, the domain-unaffected areas of the bilayer become significantly more ordered, with intensity-weighted fluorescence lifetimes of BODIPY-PM shifting to about 2000 ps.

To conclude, at low concentrations in DOPC/Chol 60/40 bilayers, simvastatin merely transfers cholesterol from the bilayer into the nanoscale domains, significantly decreasing the cholesterol concentration in the bilayer, and thus making the domain-unaffected areas of the bilayer disordered and DOPC-like. At higher than 1 μM concentrations, simvastatin not only transfers the cholesterol to nanoscale domains and increases their size but also increases the microviscosity of domain-free regions of the bilayer. Consequently, simvastatin has a bimodal effect on the microviscosity and lipid order of domain-free bilayer areas. Cholesterol transfer from the bilayer into the circular domains reduces the lipid order, whereas the competing mechanism, namely, integration of simvastatin into the domain-free areas of the bilayer, increases the lipid order. Spatially uniform fluorescence lifetimes of BODIPY-PM in the domains (particularly evident after 10–100 μM addition of simvastatin), as well as uniform Cy5-Chol fluorescence intensities in the said domains, indicate that simvastatin not merely attaches to the domains at the phase separation boundaries, thereby increasing their size, but forms a single homogeneous phase with the cholesterol that is present in the domain.

To further assess the membrane-modulating effects of statins, we analyzed the electrical properties of tBLMs using electrochemical impedance spectroscopy (EIS).^41^ The EIS response can reveal structural changes in tBLMs, such as the formation of water-filled pores and nanometer-sized defects.^41^ The addition of simvastatin to DOPC and DOPC/Chol 60/40 tBLMs did not produce the characteristic EIS spectrum alterations associated with membrane damage and pore formation, indicating that phase separation boundaries are continuous with the lipid bilayer and no defects are being produced (Figure S12).

**Figure 5:**
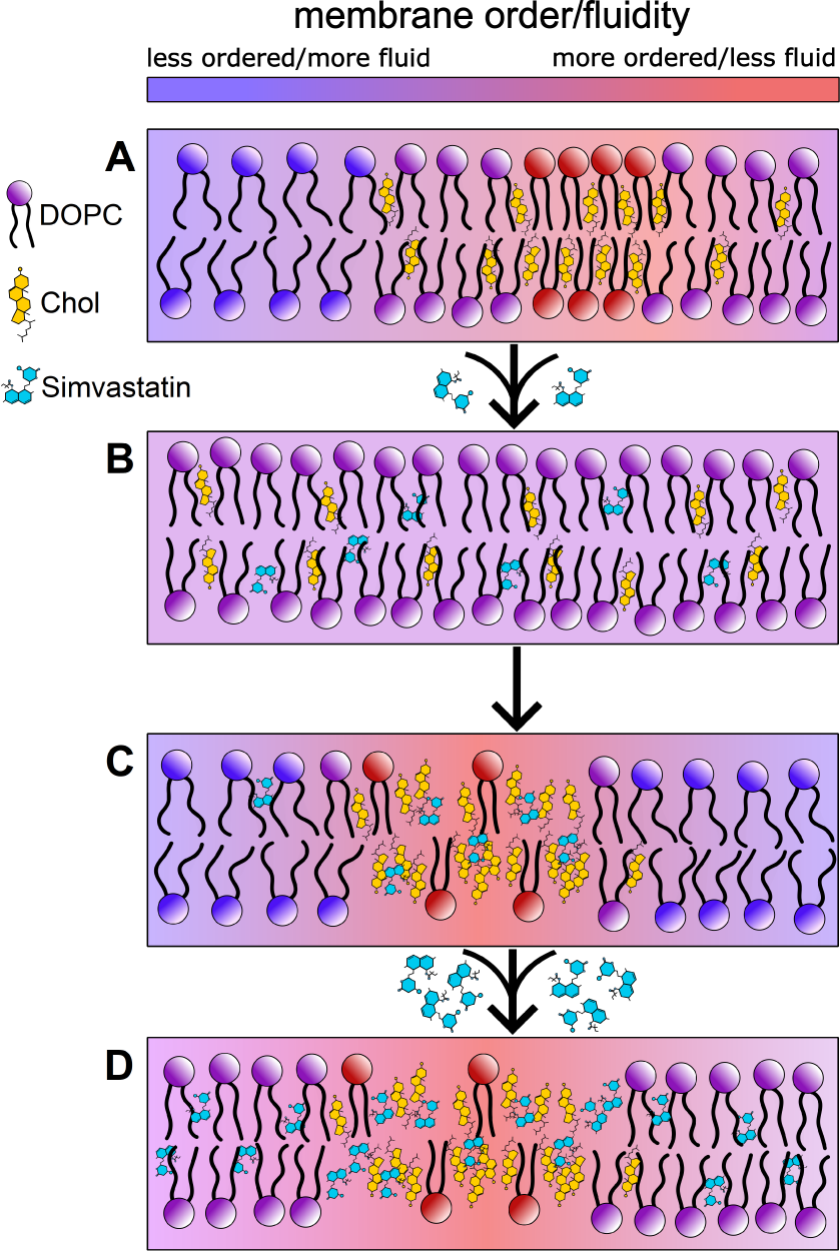
Proposed mechanism of action of simvastatin in cholesterol-rich bilayers. (A) Initial heterogeneous lipid bilayer. (B) Simvastatin-induced cholesterol transfer and homogenization of the lipid bilayer. (C) Nanoscale cholesterol-rich domain formation at the critical concentration of simvastatin. (D) Lipid ordering effect of simvastatin at concentrations above the critical concentration.

Based on our experimental results, we propose that each lipid system will likely have its own critical concentration of simvastatin, at which most of the cholesterol will be bound to the nanoscale domains (Figure 5A,5C). Moreover, if cholesterol heterogeneities are present in the bilayer, simvastatin will equilibrate the cholesterol concentration throughout the bilayer prior to domain formation (Figure 5B). Above the critical concentration, simvastatin will not only increase the size and microviscosity of the domains but also populate unoccupied cholesterol sites in the bilayer and increase the order of lipids, as is observed in pure DOPC bilayers and in DOPC/Chol 60/40 bilayers at higher simvastatin concentrations (Figure 5D). Most likely, the critical simvastatin concentration will be different for various lipid bilayer compositions and cholesterol concentrations; thus, the same concentration of simvastatin may result in different lipid-bilayer modulating effects, depending on the bilayer or the plasma membrane composition of the live cell.

Finally, we discovered that simvastatin-induced circular domains can mix into a single phase with the domain-free areas of the lipid bilayer when DOPC/Chol 60/40 tBLMs are being laser-heated (Figures S10 and S11). When circular domains are laser-irradiated during their formation, the intensity-weighted fluorescence lifetimes of BODIPY-PM gradually decrease within the circular domains, whereas the domain-surrounding areas of the lipid bilayer become more ordered and display increased intensity-weighted fluorescence lifetimes of BODIPY-PM. When tBLMs are allowed to cool to room temperature, the increased intensity-weighted fluorescence lifetimes of BODIPY-PM decrease in the domain-unaffected areas of the lipid bilayer, and the domains themselves become more ordered (Figure S11). Additionally, certain circular domains vanish altogether upon laser irradiation and do not reappear in the same area once the lipid bilayer has cooled to room temperature (Figures S10 and S11). At higher temperatures, simvastatin and cholesterol-rich circular domains can form a single phase with the lipid bilayer, releasing cholesterol and simvastatin into the domain-surrounding areas of the lipid bilayer, resulting in enhanced local microviscosity. We hypothesize that at physiological temperatures, depending on the bilayer lipid composition and the concentration of simvastatin, simvastatin-induced phase separation may be reduced with some circular domain mixing or partially mixing with the lipid bilayer, thus increasing the local microviscosity. We speculate that once simvastatin integrates into the lipid bilayer, cholesterol may provide additional stabilization to the amphiphilic simvastatin molecules through the formation of hydrogen or van der Waals bonds, thereby forming transient complexes. These cholesterol–simvastatin complexes might disrupt the initial bonding between cholesterol and the surrounding lipids in the lipid bilayer, enhancing cholesterol mobility and initiating homogenization of the lipid bilayer. The subsequent clustering of cholesterol–simvastatin complexes into the circular domains likely provides further stabilization as multiple cholesterol molecules may interact electrostatically with simvastatin. In support of this hypothesis, our experimental results show that the phase separation between circular domains and domain-free phases of the lipid bilayer decreases with increasing temperature (Figure S10). This domain-diminishing effect with an increase in temperature likely results from an increase in the entropy of mixing (-*T*Δ*S*), which overcomes the positive enthalpy of mixing (Δ*H*) and leads to an overall negative free energy for the phase mixing process at elevated temperatures. The positive enthalpy of mixing (Δ*H*) is likely a result of the presence of multiple weak bonds between simvastatin and cholesterol in the phase-separated domains.

### 2.2 Imaging of Statin-Induced Microviscosity Changes in Live Cells

**Figure 6:**
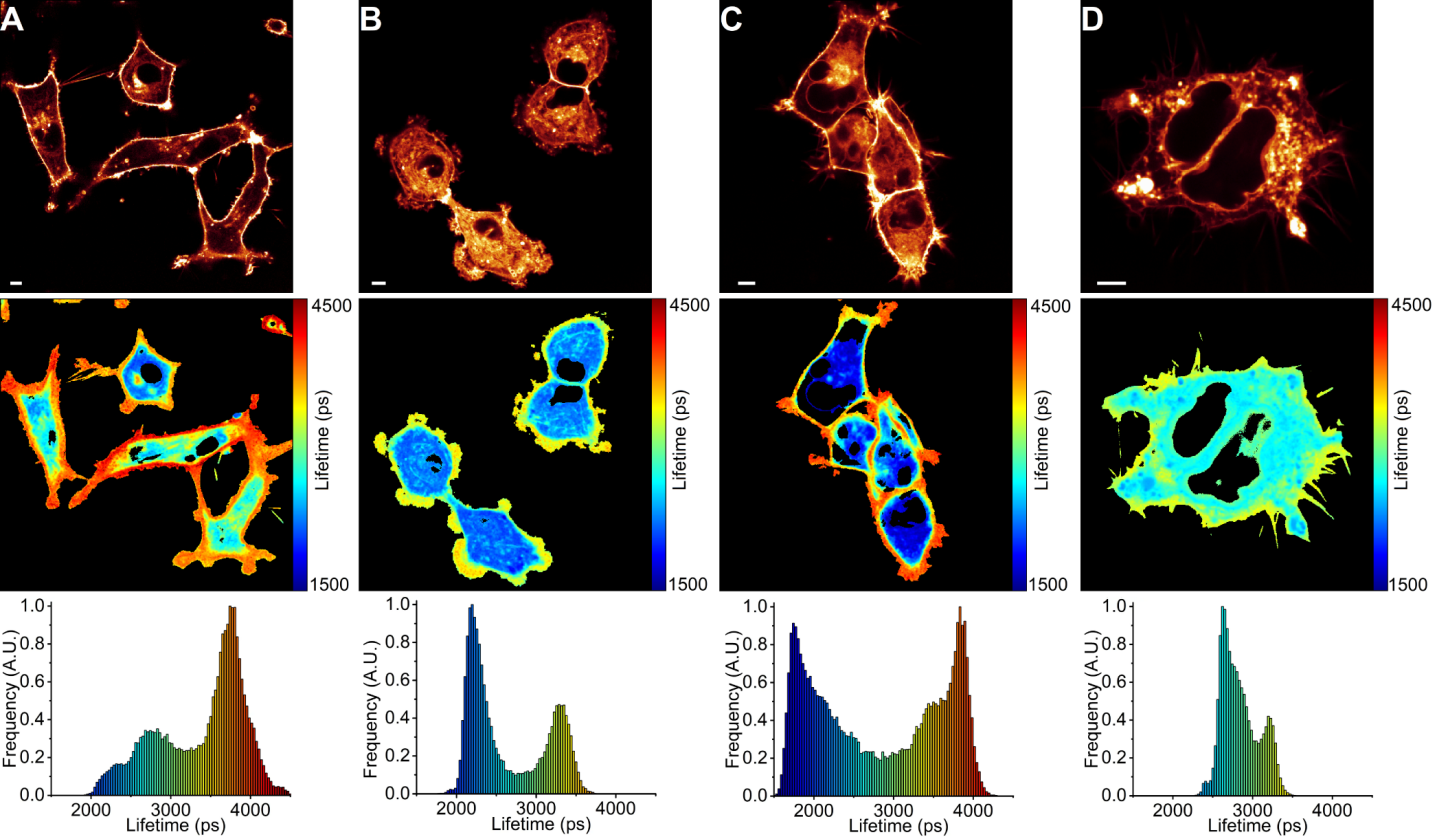
FLIM of BODIPY-PM in live A549 and HEK 293T cells: (A) A549 cells before simvastatin addition. (B) A549 cells 5 min after simvastatin addition. (C) HEK 293T cells before simvastatin addition. (D) HEK 293T cells 5 min after simvastatin addition. The top panel shows images of fluorescence intensity. FLIM images are shown in the middle panel. The corresponding lifetime histograms are shown in the bottom panel. Scale bars are 5 μm.

**Figure 7:**
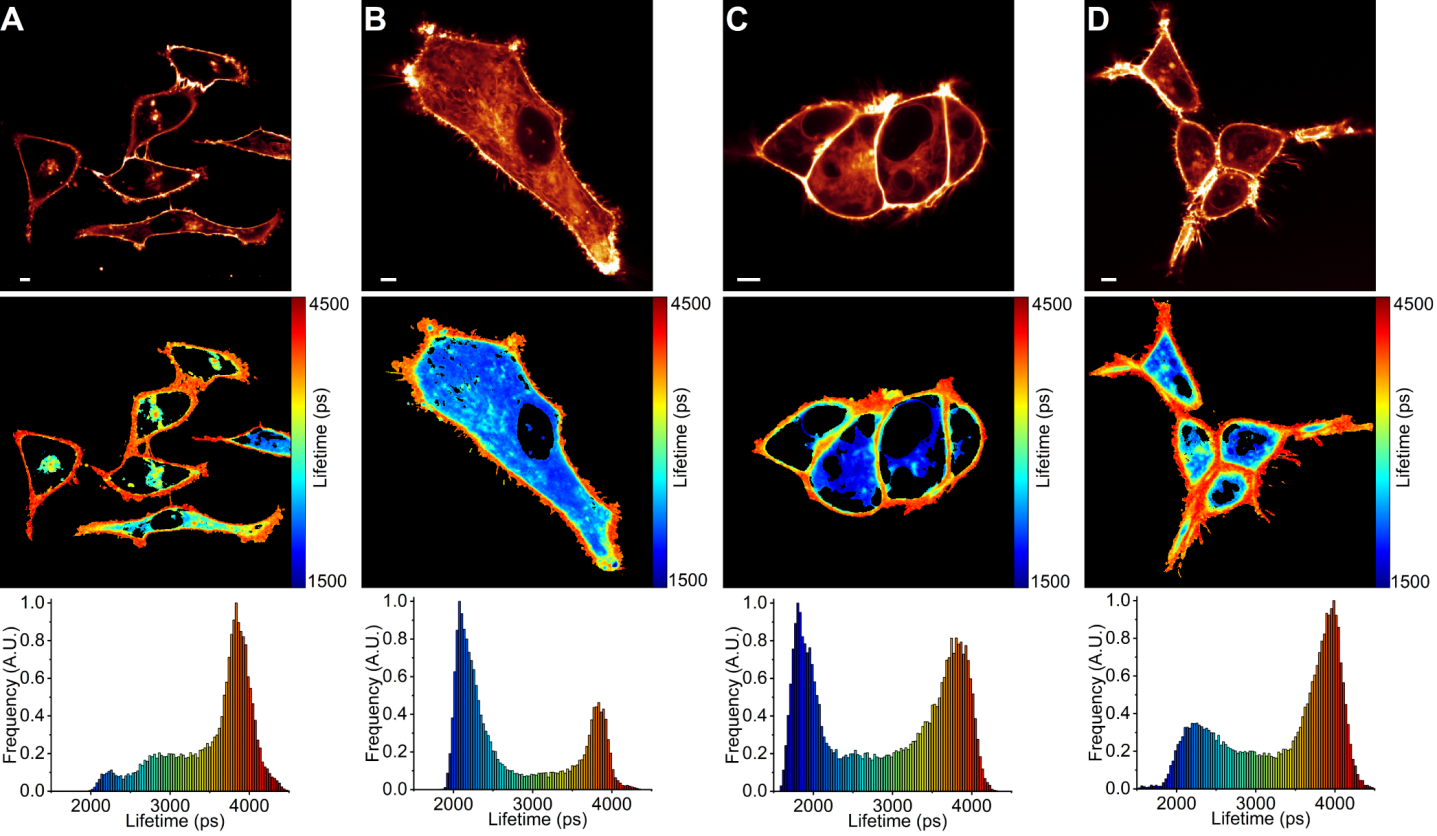
FLIM of BODIPY-PM in live A549 and HEK 293T cells. (A) A549 cells 5 min after pravastatin addition. (B) A549 cells 4 h after simvastatin addition. (C) HEK 293T cells 5 min after pravastatin addition. (D) HEK 293T cells 4 h after simvastatin addition. The top panel shows images of fluorescence intensity. FLIM images are shown in the middle panel. The corresponding lifetime histograms are shown in the bottom panel. Scale bars are 5 μm.

Next, we investigated the effects of statins on the plasma membranes of live lung cancer (A549) and human immortalized kidney (HEK 293T) cells stained with BODIPY-PM. In contrast to model lipid bilayer membranes, live cell membranes are significantly more ordered and include a diverse range of lipids, with cholesterol accounting for around 30–40% of the total membrane lipid content.^42^ Curiously, the addition of simvastatin (10 μM) to live A549 and HEK 293T cells did not lead to the formation of circular domains in the plasma membrane. Instead, simvastatin, within 5 min, significantly reduced the microviscosity of the plasma membranes in both A549 and HEK 293T cells, with intensity-weighted fluorescence lifetimes of BODIPY-PM decreasing from about 3750 ps in intact A549 and HEK 293T cells to 3250 and 3100 ps in simvastatin-affected A549 and HEK 293T cells, respectively (Figure 6). A significant decrease in BODIPY-PM intensity-weighted fluorescence lifetimes after simvastatin addition corresponds to a reduction of plasma membrane microviscosity values (in methanol–glycerol calibration mixtures) from 370 cP (in intact A549/HEK 293T cells) to 280 and 260 cP in A549 and HEK 293T cells, respectively. We also note that following simvastatin treatment, the cells temporarily lost their normal morphology and became more rounded, although the plasma membrane integrity remained intact (Figure 6B,D). We suspect that cellular shape alterations are caused by rapidly decreasing lipid order in plasma membranes, as comparable morphological changes occur when plasma membranes are rapidly cholesterol-enriched or depleted.^43,44^ Moreover, after 15 min, the cells relax to their normal morphologies, while maintaining decreased plasma membrane microviscosity values (Figure S13). We also discovered that the fluorescence intensity of BODIPY-PM in the plasma membrane is greatly reduced after simvastatin addition, while the fluorescence intensity in the cytoplasm increases (Figure 6B,D). Furthermore, following simvastatin addition, brightly fluorescent, micrometer-sized circular vesicle-like structures appear in the cytoplasm of live cells (Figures 6 and S13). Since BODIPY-PM is an anionic lipophilic probe with high affinity for the plasma membrane and slow internalization rates, we suspect that the simultaneous decrease in fluorescence intensity in the plasma membrane and the appearance of bright vesicle-like structures in the cytoplasm implies that simvastatin promotes membrane internalization, which leads to the depletion of the probe in the plasma membrane and the accumulation of the probe in the cytoplasm. We hypothesize that the decrease in the microviscosity of plasma membranes and the absence of highly viscous domains following simvastatin treatment might be attributed to rapid internalization of simvastatin-induced domains, especially since the formation of cholesterol-rich domains occurs on the time scale of minutes. As a result, cells may internalize simvastatin-affected cholesterol-rich regions to restore the normal lipid order in the plasma membrane, thereby reducing the cholesterol concentration in the bilayer, which leads to decreased plasma membrane microviscosities. In contrast to simvastatin, the addition of pravastatin (10 μM) did not affect the microviscosities of plasma membranes in either A549 or HEK 293T cells (Figure 7A,C). We thus conclude that pravastatin has no effect on the microviscosities of either cellular plasma membranes or model lipid bilayers, most likely due to its hydrophilic nature and inability to integrate into lipid bilayers.

We also noticed that simvastatin-induced plasma membrane fluidization is a temporary effect since cells treated with simvastatin for 4 h completely revert to their initial plasma membrane microviscosity values (Figure 7B,D). Finally, we investigated the influence of simvastatin-fluidized membranes on the intracellular chemotherapeutic drug doxorubicin accumulation in A549 cells. In accordance with previous FLIM measurements, A549 cells treated for 5 min with simvastatin displayed about 2-times higher doxorubicin fluorescence intensities compared to untreated, pravastatin-treated (5 min and 4 h), or 4 h simvastatin-treated A549 cells (Figure S14 and S15).

Since the diffusion of molecules across the cell membrane is viscosity-proportional,^45^ simvastatin-like compounds that fluidize lipid bilayers may be useful in cancer therapy to permeabilize the cell membranes and allow for enhanced drug uptake.^46,47^ Despite the fact that simvastatin fluidizes the plasma membranes of both malignant (A549) and nonmalignant (HEK 293T) cells equally well, the use of tumor-specific nanoparticles^48^ or liposomes^49^ in combination with simvastatin may improve drug absorption or eliminate the drawbacks of poorly cell-penetrating nanoparticles.^50^

## 3 Conclusions

In conclusion, we have explored the bilayer-modulatory mechanisms of simvastatin and pravastatin in model lipid bilayers and live cell plasma membranes. Our results demonstrate that pravastatin does not change the microviscosities of model lipid bilayers or live cell plasma membranes. In contrast, we show that simvastatin is capable of forming viscous cholesterol-rich nanoscale circular domains in cholesterol-rich lipid bilayers. We demonstrate that simvastatin-induced nanodomains are composed of both cholesterol and simvastatin, with the size and microviscosity of the domains being dependent on the simvastatin concentration. Furthermore, simvastatin has a bimodal effect on the microviscosities of cholesterol-rich lipid bilayers, which manifests with a simultaneous decrease in the microviscosity of the lipid bilayer due to the binding of cholesterol into the nanoscale domains and an increase in the microviscosity due to the integration of simvastatin into the domain-unaffected areas of the lipid bilayer. Additionally, we provide a mechanistic model for explaining the bimodal effects of hydrophobic statins on model lipid bilayers. Moreover, we show that simvastatin has a distinct function on live cell plasma membranes, unlike in model lipid bilayers: instead of forming viscous nanodomains, simvastatin uniformly reduces the microviscosity of plasma membranes without affecting their integrity. In addition, the fluidizing effects of simvastatin on live cell plasma membranes are temporal, and within 4 h, the plasma membranes return to their initial microviscosity values. Finally, our results demonstrate that simvastatin-affected plasma membranes are more permeable to chemotherapeutic drugs.

## 4 Experimental Section

### 4.1 Dyes, Reagents, and Lipids

Stock solutions of 1 mM BODIPY-PM were prepared in water and diluted for further experiments in tBLMs and live cells. DOPC, cholesterol, and Cy5-cholesterol were obtained from Avanti Polar Lipids. Doxorubicin, simvastatin, and pravastatin were obtained from Sigma-Aldrich, and 10 mM stock solutions of doxorubicin, simvastatin, and pravastatin were prepared in DMSO and diluted for subsequent experiments.

### 4.2 Formation of Tethered Bilayer Lipid Membranes (tBLMs)

For the formation of tBLMs, clean glass slides were first coated with a 100 nm layer of gold by magnetron sputtering using a PVD75 system (Kurt J. Lesker Co). Gold-coated slides were then immersed overnight in a solution of 20-tetradecyloxy-3,6,9,12,15,18,22-heptaoxahexatricontane-1-thiol (WC14) as an anchoring molecule and β-mercaptoethanol in a molar ratio of 3:7, and a total thiol concentration of 0.05 mM to form a self-assembled monolayer (SAM). The lipid bilayer was then formed by the multilamellar vesicle (MLV) fusion method. Briefly, stock chloroform solutions of lipids were mixed in appropriate ratios and placed under gentle nitrogen stream for at least 2 h. Phosphate buffer solution with pH 4.5 was added on top of the dried lipid film, and the mixture was slowly resuspended, forming an MLV solution (1 mM total lipid concentration). Vesicle solution was added on the SAM-modified gold surface, and membranes were allowed to form for 1 h before rinsing them using PBS with pH 7.1. For BODIPY-PM staining, prepared membranes were incubated in an aqueous solution with the dye (1 μM) for 3 min, and then washed with PBS to remove the unincorporated dye.

### 4.3 Imaging of Live Cells

Cell imaging experiments were performed using the human lung cancer A549 and immortalized human embryonic kidney HEK 293T cell lines (ATCC). The cells were cultured in Dulbecco’s Modified Eagle‘s Medium (DMEM) supplemented with 10% fetal bovine serum (FBS), 100 IU/mL penicillin, and 100 μg/mL streptomycin (Thermo Fisher). The cells were incubated at 37 °C with 5% CO_2_. Before imaging, the cells were seeded into ibidi μ-dish (ibidi) at a seeding density of 10 000 cells/mL and allowed to grow for 24 h. For cell imaging, 1 μM BODIPY-PM solution (in water) was added to the culture medium for 5 min at 37 °C. Live cells were treated with statins at 37 °C. FLIM imaging was done at room temperature using Leica SP8 with 63× objective (HC PL APO oil immersion, N.A. −1.4, Leica).

### 4.4 Data Analysis

FLIM images were analyzed with FLIMFIT software (v4.6.1, Imperial College London). The biexponential fluorescence decay model with intensity-weighted mean lifetimes (eq 1) was applied for FLIM measurements:
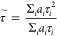
1where *a*_*i*_ and τ_*i*_ are the amplitudes of the individual components. The goodness-of-fit parameter (χ^2^) was 1.3 or less.

### 4.5 Electrochemical Impedance Spectroscopy (EIS)

Electrochemical impedance was measured using a PalmSens4 potentiostat (PalmSens), controlled by PSTrace 5.9 software. The measurements were carried out in the frequency range between 0.1 Hz and 100 kHz with 10 logarithmically distributed measurement points per decade, and a perturbation amplitude of 10 mV. A three-electrode system was used, with a gold coated glass slide as a working electrode, a saturated silver–silver chloride Ag/AgCl/NaCl (aq. sat.) microelectrode as a reference electrode and a platinum wire as a counter electrode. Results were normalized to a geometric surface area.

## Supporting Information Available

Simvastatin-induced microviscosity changes (Figures S1 and S4); time-series of BODIPY-PM fluorescence intensity images (Figures S2 and S5); effects of pravastatin on DOPC/Chol 60/40 tBLMs (Figures S3); fluorescence intensity images of Cy5-cholesterol and doxorubicin (Figures S6 and S14); effects of various simvastatin concentrations on the microviscosity of DOPC/Chol tBLMs (Figures S7–S9); circular domain formation with a laser-heated area (Figure S11); EIS spectra of tBLMs affected by simvastatin (Figure S12); FLIM images of BODIPY-PM (Figure S13); doxorubicin fluorescence intensity images in statin-affected live cells (Figure S15) (PDF)
