# Supplementary material for: Influence of Simvastatin and Pravastatin on the Biophysical Properties of Model Lipid Bilayers and Plasma Membranes of Live Cells
Source: ACS Biomater Sci Eng. 2024 Aug 24;10(9):5714–22. doi: 10.1021/acsbiomaterials.4c00911 (PMC11388144; doi:10.1021/acsbiomaterials.4c00911)
Supplement: Supplementary file 1 — ab4c00911_si_001.pdf [file ab4c00911_si_001.pdf]

Supporting Information for:

Influence of simvastatin and pravastatin on the biophysical properties of model lipid bilayers and plasma membranes of live cells

Artūras Polita,*[a] Rūta Bagdonaitė,[a] Shivabalan Arun Prabha,[a] and Gintaras Valinčius [a]

[a.] *Life Sciences Center, Institute of Biochemistry, Vilnius University, Saulėtekio av. 7, Vilnius, LT-10257, Lithuania. E-mail: arturas.polita@gmc.vu.lt.*

Table of Contents

**Figure S1**	Simvastatin-induced microviscosity changes to homogenous areas of DOPC/Chol 60/40 tBLMs
**Figure S2**	Time-series of BODIPY-PM fluorescence intensity images acquired after addition of simvastatin
**Figure S3**	Effects of pravastatin on DOPC/Chol 60/40 tBLMs
**Figure S4**	Simvastatin-induced microviscosity changes to heterogeneous areas of DOPC/Chol 60/40 tBLMs
**Figure S5**	Time-series of BODIPY-PM fluorescence intensity images
**Figure S6**	Fluorescence intensity images of Cy5-Cholesterol in DOPC/Chol 60/40 tBLMs
**Figure S7**	1 µM Simvastatin-induced microviscosity changes to DOPC/Chol 60/40 tBLMs
**Figure S8**	20 µM Simvastatin-induced microviscosity changes to DOPC/Chol 60/40 tBLMs
**Figure S9**	100 µM Simvastatin-induced microviscosity changes to DOPC/Chol 60/40 tBLMs
**Figure S10**	Time-series of BODIPY-PM FLIM images acquired with laser-heated area after 5 µM simvastatin addition
**Figure S11**	Circular domain formation with laser-heated area after 5 µM simvastatin addition
**Figure S12**	Electrochemical Impedance (EIS) spectra of DOPC and DOPC/Chol 60/40 tBLMs upon exposure to simvastatin and pravastatin
**Figure S13**	FLIM of BODIPY-PM in HEK 293T cells 15 min after simvastatin addition
**Figure S14**	Fluorescence intensity images of doxorubicin in A549 cells
**Figure S15**	Mean doxorubicin fluorescence intensities in A549 cells with different simvastatin or pravastatin treatments

Simvastatin-induced microviscosity changes to homogenous areas of DOPC/Chol 60/40 tBLMs

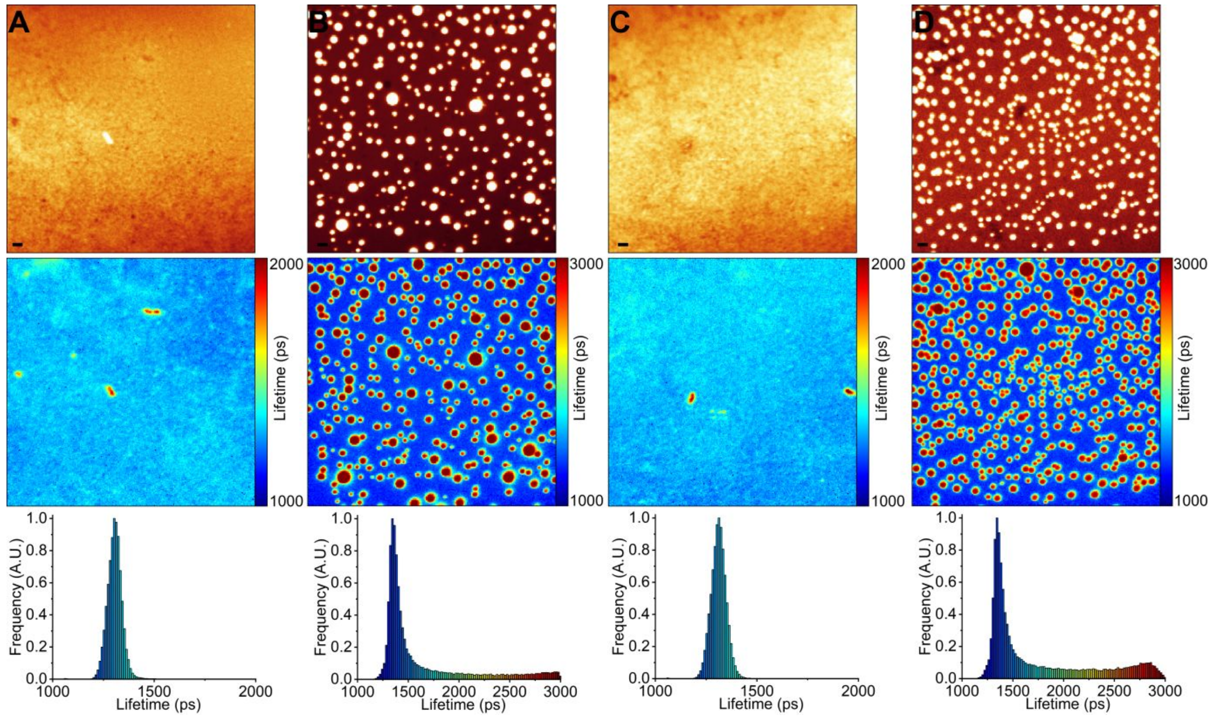

Figure S1. FLIM of BODIPY-PM in DOPC/Chol 60/40 tBLMs. (A, C) tBLMs before simvastatin addition. (B, D) tBLMs 5 min after addition of simvastatin (10 µM). The top panel shows images of fluorescence intensity. FLIM images are shown in the middle panel. The corresponding lifetime histograms are shown in the bottom panel. Scale bars are 1 µm.

We performed FLIM of BODIPY-PM in highly homogeneous areas of DOPC/Chol 60/40 tBLMs to investigate whether the size, number, and most importantly, microviscosity of simvastatin-induced domains are similar to heterogeneous regions of tBLMs (Fig. S1). The addition of simvastatin (10 µM) to DOPC/Chol 60/40 tBLMs led to the formation of circular domains (Fig. S1B and S1D). We did not observe any significant differences in the size, number, and microviscosity of simvastatin-induced domains between highly homogeneous and heterogeneous areas of DOPC/Chol tBLMs. Moreover, we have imaged the fluorescence intensity of BODIPY-PM in area S1C throughout the formation of circular domains (Fig. S2).

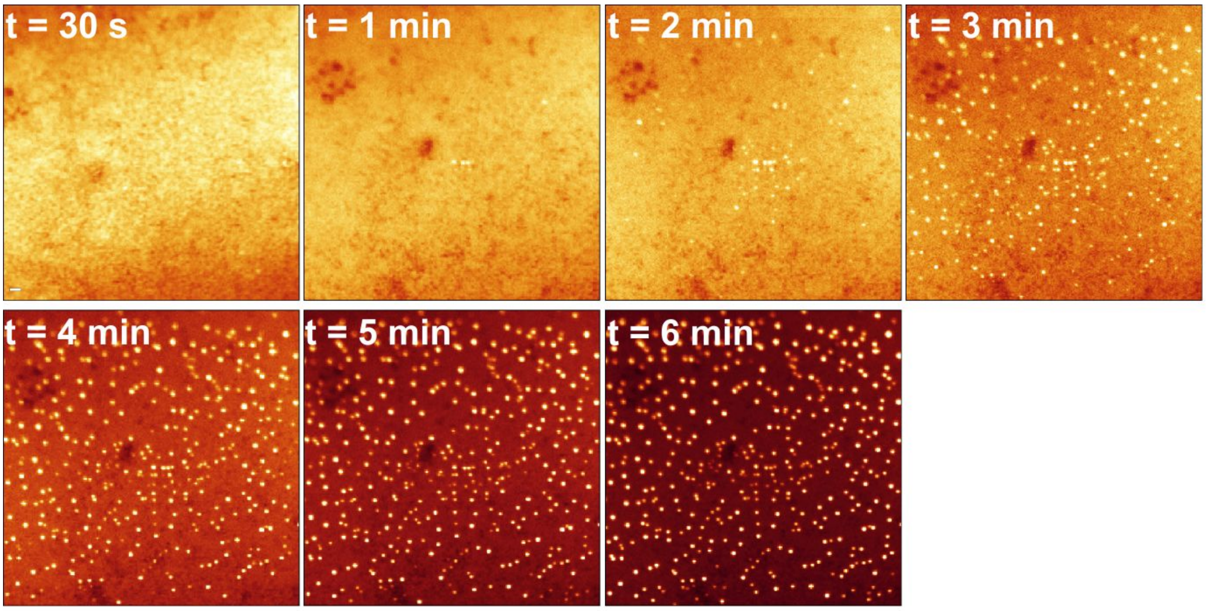

Figure S2. Time-series of BODIPY-PM fluorescence intensity images acquired after addition of simvastatin in area S1C. Scale bar is 1 µm.

The majority of the domains form three to four minutes after simvastatin addition. Furthermore, simvastatin-induced domains appear to be immobile (Fig. S2).

Effects of pravastatin on DOPC/Chol 60/40 tBLMs

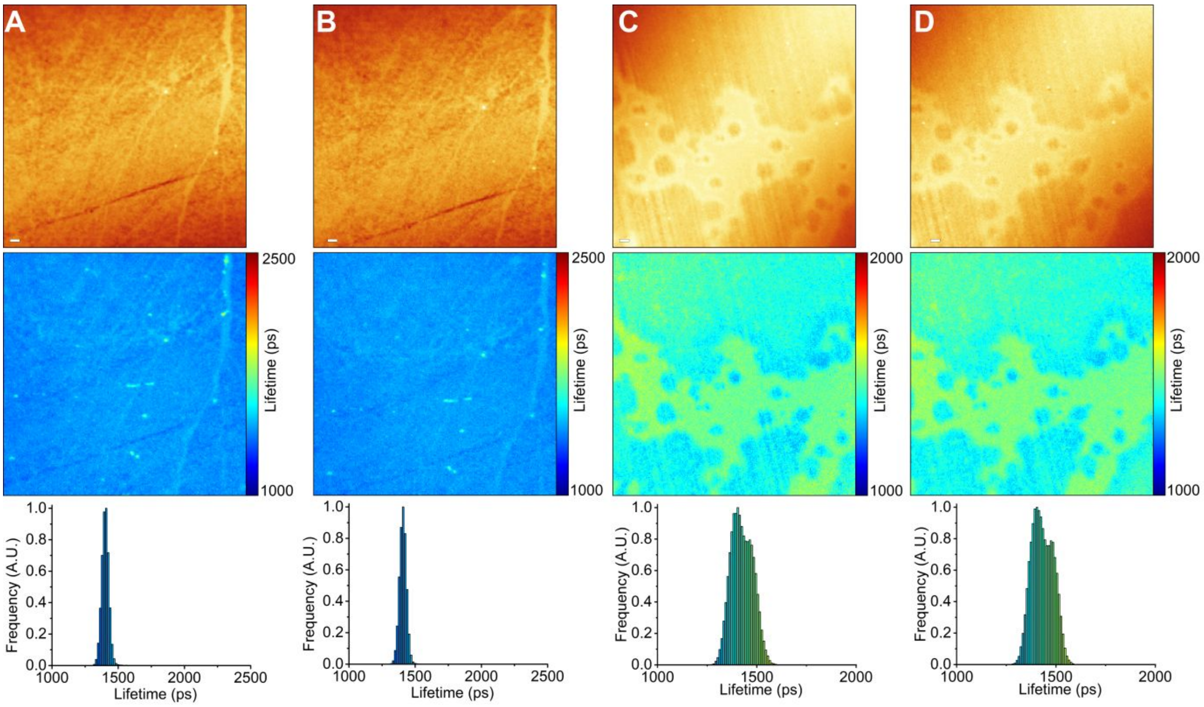

Figure S3. FLIM of BODIPY-PM in DOPC/Chol 60/40 tBLMs. (A, C) tBLMs before pravastatin (10 µM) addition. (B, D) tBLMs 5 min after addition of pravastatin (10 µM). The top panel shows images of fluorescence intensity. FLIM images are shown in the middle panel. The corresponding lifetime histograms are shown in the bottom panel. Scale bars are 1 µm

To investigate the effects of pravastatin on the microviscosity of tBLMs, we performed FLIM measurements of DOPC/Chol 60/40 bilayers stained with BODIPY-PM. The addition of pravastatin (10 µM) produced no microviscosity changes nor induced the formation of circular domains in either homogeneous or heterogeneous areas of tBLMs (Fig. S3). We suspect that pravastatin is too hydrophyllic, compared to simvastatin, to integrate into the lipid bilayer and cause microviscosity alterations.

Simvastatin-induced microviscosity changes to heterogeneous areas of DOPC/Chol 60/40 tBLMs

To investigate the cholesterol transfer effects of simvastatin in DOPC/Chol 60/40 tBLMs, we performed both the initial and final FLIM measurements of BODIPY-PM before and after simvastatin addition on DOPC/Chol 60/40 tBLMs (Fig. S4), as well as time-series measurements of BODIPY-PM fluorescence intensity during the domain formation period (Fig. S5).

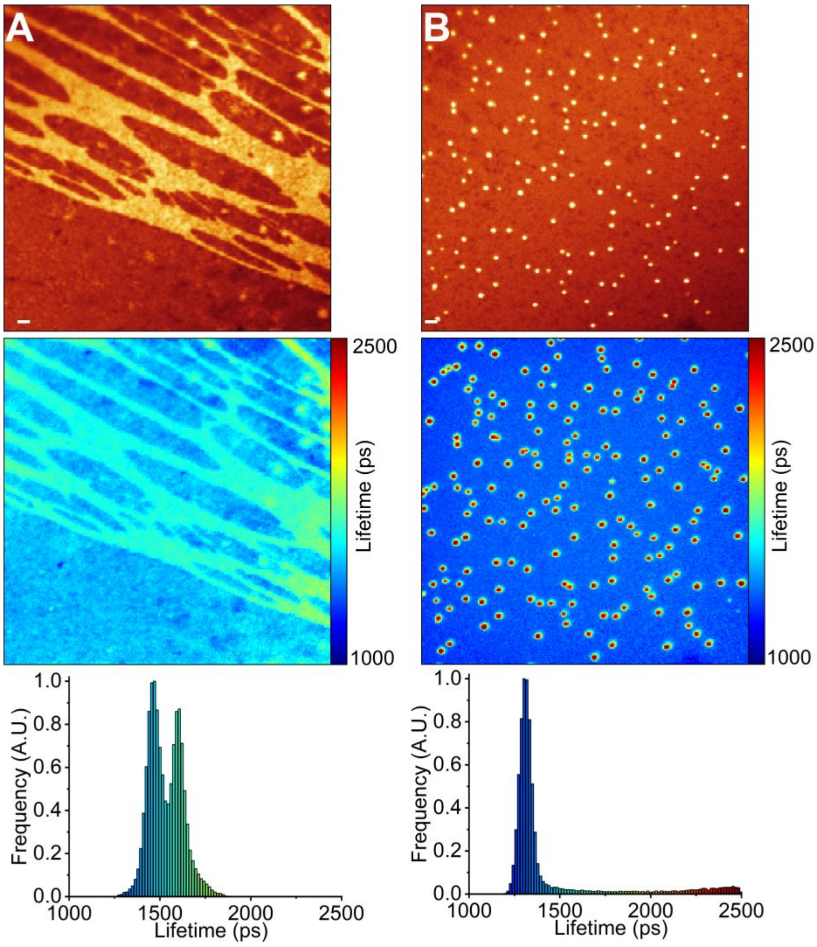

Figure S4. FLIM of BODIPY-PM in heterogneous DOPC/Chol 60/40. (A) tBLM before simvastatin (10 µM) addition. (B) tBLM 5 min after simvastatin addition. The top panel shows images of fluorescence intensity. FLIM images are shown in the middle panel. The corresponding lifetime histograms are shown in the bottom panel. Scale bars are 1 µm.

After the addition of simvastatin (10 µM), cholesterol-rich regions disappear and circular nanoscale domains begin to form (Fig. S4B). We, again, did not observe any movement of said domains in either

their formation period or after the domains were fully formed. Additionally, we provide a full-time series of fluorescence intensity images of BODIPY-PM during domain formation (Fig. S5).

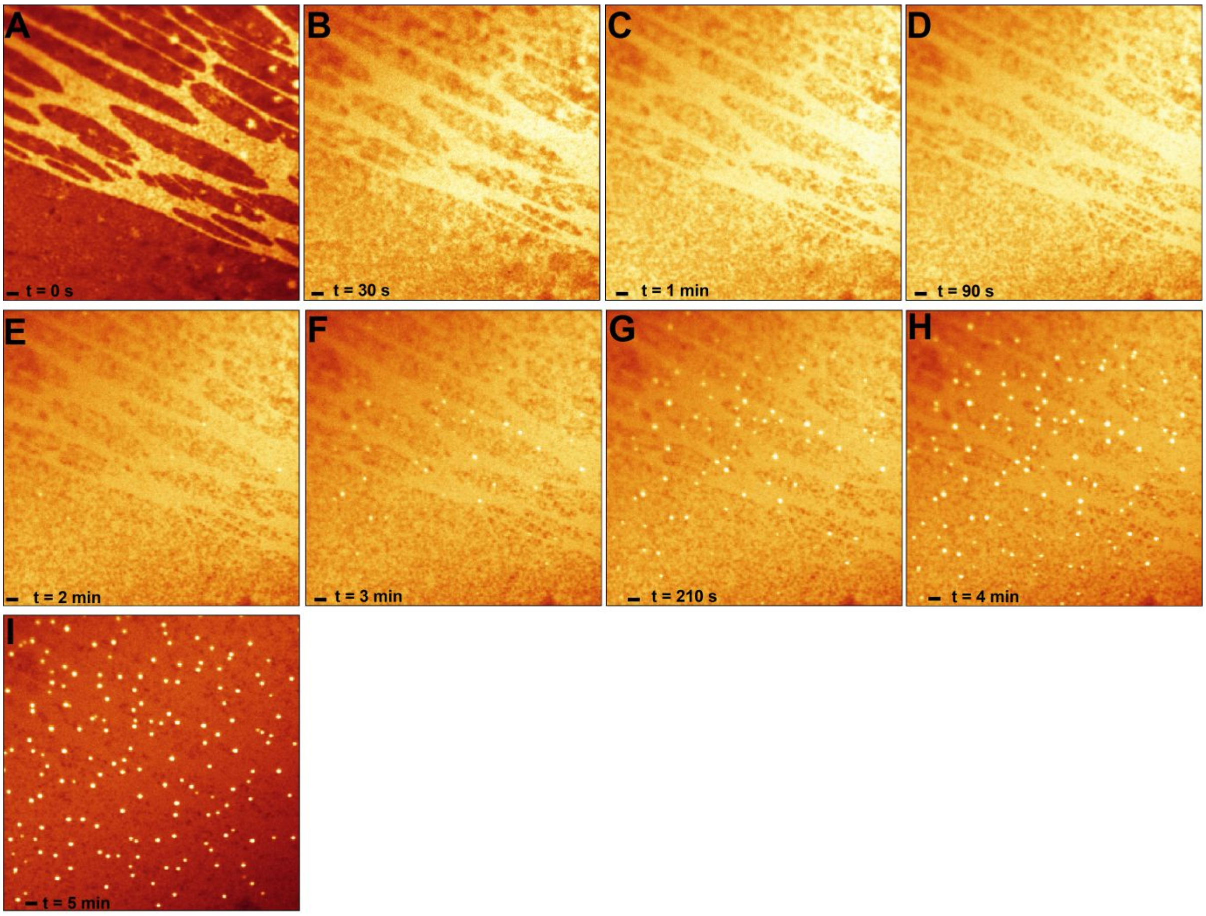

Figure S5. Time-series of BODIPY-PM fluorescence intensity images. (A) before addition of simvastatin. (B-I) fluorescence intensity images after simvastatin addition. Time-stamps indicate image acquisition time after simvastatin addition. Scale bars are 1 μm.

After the addition of simvastatin, cholesterol-rich regions begin to dissipate and the fluorescence intensities of BODIPY-PM begin to equilise throughout the bilayer, indicating that simvastatin induces the homogenization of the lipid bilayer prior to domain formation (Fig. S2B-D). Since cholesterol is the main component in the binary DOPC/Chol lipid system that is responsible for the heterogeneities and higher microviscosity areas in tBLMs, we speculate that simvastatin is capable of transferring the cholesterol and equilizing its concentration in the bilayer.

Fluorescence intensity images of Cy5-Cholesterol in DOPC/Chol 60/40 tBLMs

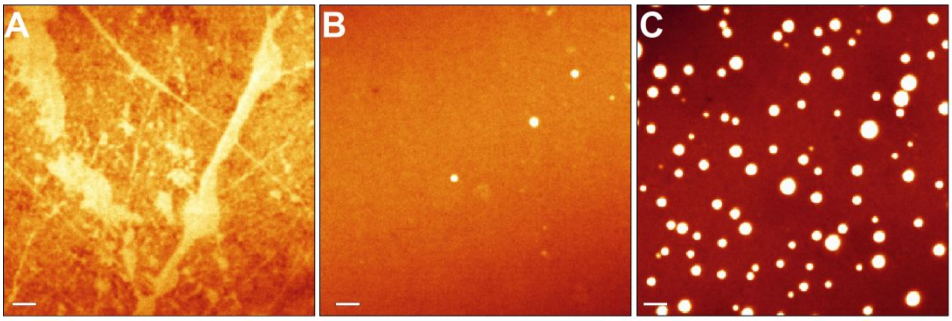

Figure S6. Fluorescence intensity images of Cy5-Chol in DOPC/Chol 60/40 tBLMs. (A) before simvastatin addition. (B) 3 min after simvastatin addition. (C) 5 min after simvastatin addition. Scale bars are 1 µm.

To investigate the composition of simvastatin-induced domains and to verify that simvastatin truly homogenizes cholesterol in the lipid bilayers, we labeled 0.25% of the cholesterol in DOPC/Chol 60/40 tBLMs with Cyanine-5-cholesterol (Fig. S6). The addition of simvastatin (10 µM) led to quick homogenization of cholesterol concentration (Fig. S6B) and the disappearance of high-intensity cholesterol-rich areas. Moreover, simvastatin-induced domains displayed about 30–40 times higher fluorescence intensities compared to domain-unaffected areas, thus indicating that greater amounts of cholesterol are present in the domains compared to the surrounding bilayer (Fig. S6C).

1 µM Simvastatin-induced microviscosity changes to DOPC/Chol 60/40 tBLMs

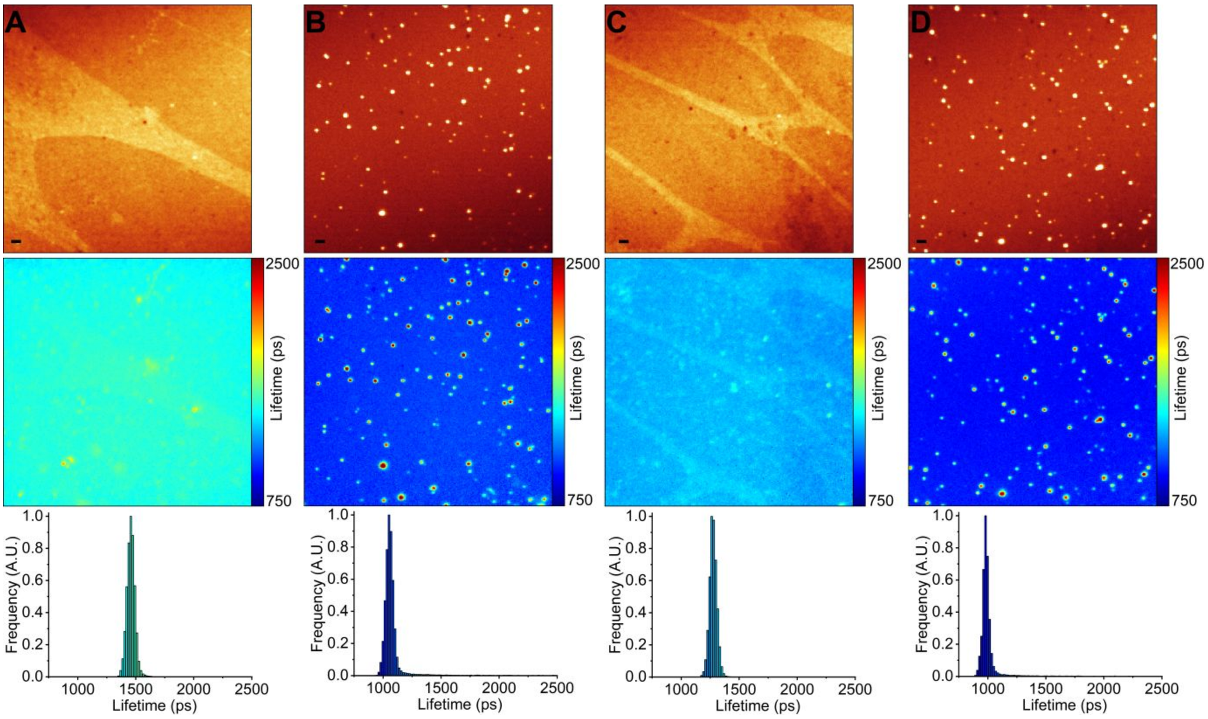

Figure S7. FLIM of BODIPY-PM in DOPC/Chol 60/40 tBLMs. (A, C) tBLMs before simvastatin (1 µM) addition. (B, D) tBLMs 5 min. after addition of simvastatin (1 µM). The top panel shows images of fluorescence intensity. FLIM images are shown in the middle panel. The corresponding lifetime histograms are shown in the bottom panel. Scale bars are 1 µm.

To investigate the influence of simvastatin concentration on the size and microviscosity of simvastatin-induced domains, we performed FLIM measurements of DOPC/Chol 60/40 tBLMs stained with BODIPY-PM and varied simvastatin concentration. The addition of simvastatin (1 µM) to DOPC/Chol tBLMs resulted in the formation of nanoscale domains with BODIPY-PM displaying intensity-weighted fluorescence lifetimes of about 2000–2500 ps, corresponding to the microviscosity values of 110–170 cP in methanol-glycerol calibration mixtures (Fig. S7). Moreover, the domains range in size from about 250 to 400 nm. We suspect that our observed size is limited by diffraction, and the real size of the domains may be much smaller. We also note that even after 1 µM of simvastatin addition, cholesterol-rich regions of tBLMs disappear (Fig. S7), and the intensity-weighted fluorescence lifetimes of BODIPY-PM shift from about 1500–1300 ps in the intact bilayer (Fig. S7A and S7C) to about 900 ps in domain-unaffected areas after simvastatin addition (Fig. S7B and S7D).

20 µM Simvastatin-induced microviscosity changes to DOPC/Chol 60/40 tBLMs

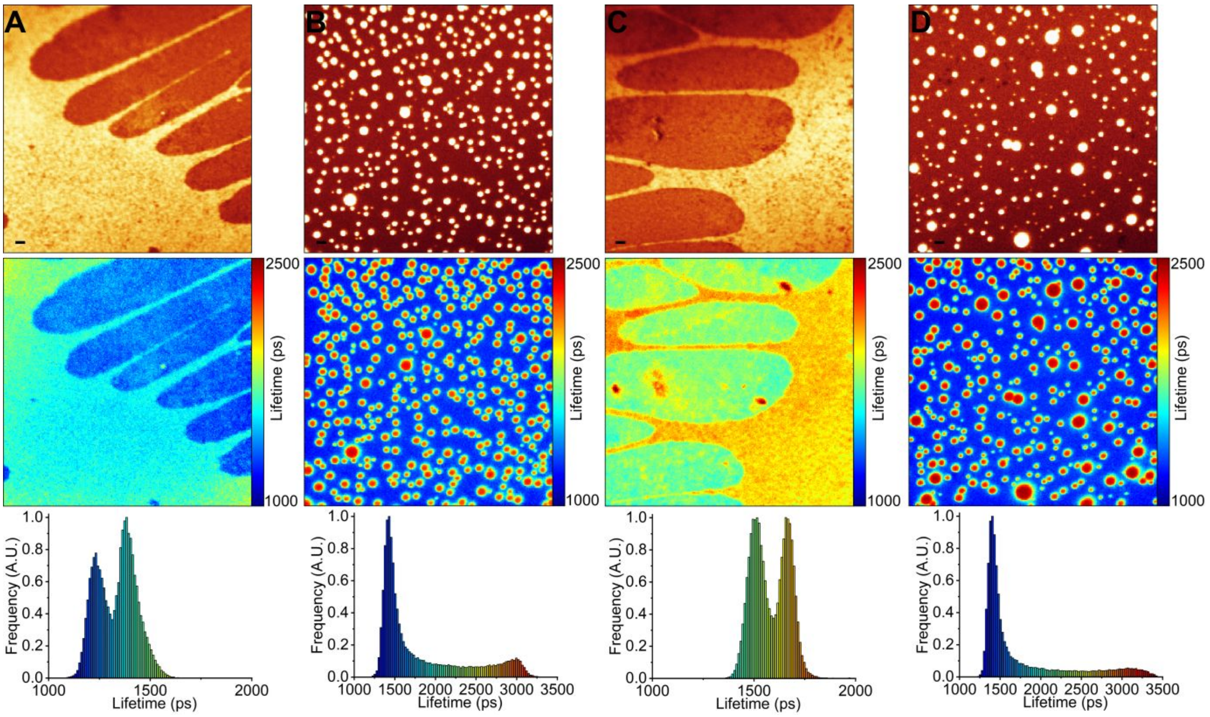

Figure S8. FLIM of BODIPY-PM in DOPC/Chol 60/40 tBLMs. (A, C) tBLMs before simvastatin (20 µM) addition. (B, D) tBLMs 5 min. after addition of simvastatin (20 µM). The top panel shows images of fluorescence intensity. FLIM images are shown in the middle panel. The corresponding lifetime histograms are shown in the bottom panel. Scale bars are 1 µm.

The addition of 20 µM of simvastatin to DOPC/Chol tBLMs resulted in the formation of circular domains with higher microviscosities and larger sizes compared to 1 µM or 10 µM simvastatin addition (Fig. S8). BODIPY-PM displayed intensity-weighted fluorescence lifetimes of about 3000–3500 ps in the domains, which correspond to the viscosity values of 250–320 cP in methanol-glycerol calibration mixtures. Additionally, the size of the domains varied from about 300 to 1200 nm. Finally, after 20 µM of simvastatin addition, the cholesterol-rich areas of tBLMs disappear, and the intensity-weighted

lifetimes of BODIPY-PM in simvastatin-unaffected areas of tBLMs shift to about 1500 ps (Fig. S8). Importantly, the areas of tBLMs that were non-viscous prior to simvastatin addition display increased BODIPY-PM fluorescence lifetimes after simvastatin addition, indicating that simvastatin itself increases the order of lipids at higher concentrations (Fig. S8A and S8B).

100 µM Simvastatin-induced microviscosity changes to DOPC/Chol 60/40 tBLMs

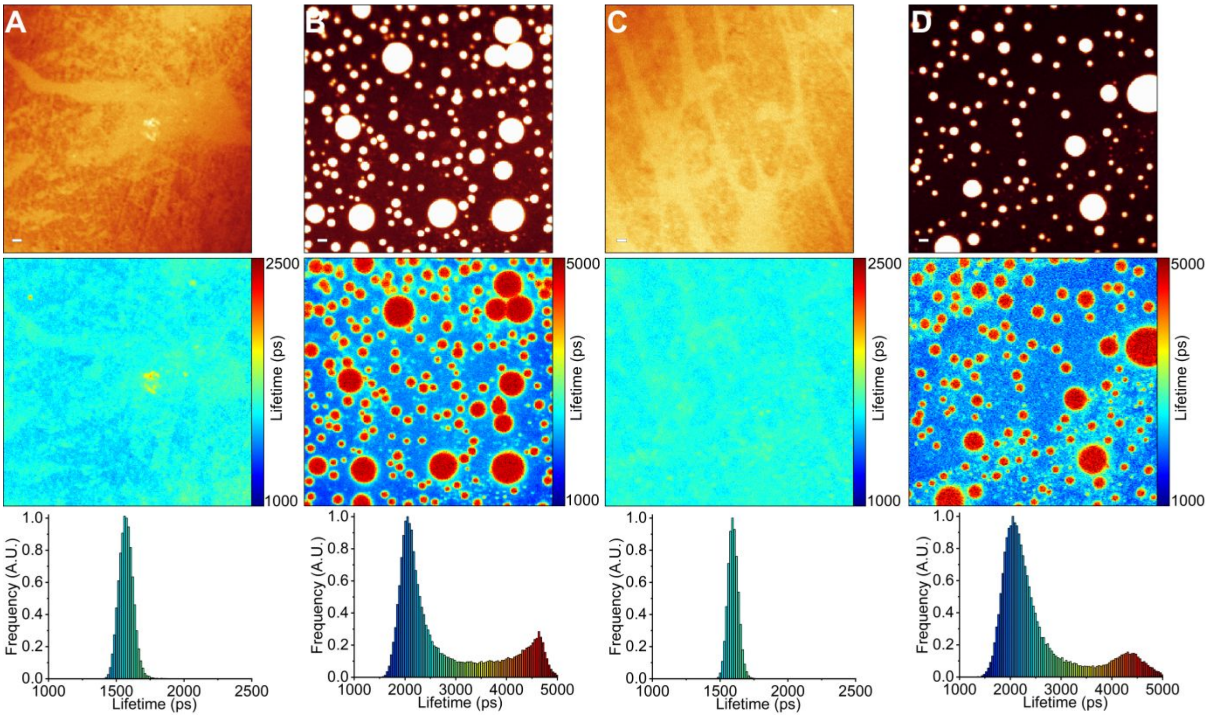

Figure S9. FLIM of BODIPY-PM in DOPC/Chol 60/40 tBLMs. (A, C) tBLMs before simvastatin (100 µM) addition. (B, D) tBLMs 5 min. after addition of simvastatin (100 µM). The top panel shows images of fluorescence intensity. FLIM images are shown in the middle panel. The corresponding lifetime histograms are shown in the bottom panel. Scale bars are 1 µm.

The addition of 100 µM simvastatin to DOPC/Chol tBLMs resulted in the formation of circular domains with very high miroviscosities and larger sizes compared to 10 µM simvastatin addition (Fig. S9). BODIPY-PM displayed intensity-weighted fluorescence lifetimes of about 4500–5000 ps in the domains, which correspond to the viscosity values of 680–880 cP in methanol-glycerol calibration mixtures. Although few nanoscale domains are present, some of the domains become micron-sized (Fig. S9). Importantly, the intensity-weighted fluorescence lifetimes of BODIPY-PM in micron-sized domains are uniform, indicating that only one phase is present in the domain, and fluorescence lifetime gradients are only observed in diffraction-limited phase borders of the domains. We also note that at 100 µM concentrations, simvastatin significantly increases the microviscosities of DOPC/Chol 60/40 tBLMs, as the intensity-weighted fluorescence lifetimes of BODIPY-PM in domain-unaffected areas of tBLMs shift from 1500 ps to 2000 ps (Fig. S9). We hypothesize that the increase in BODIPY-PM fluorescence lifetimes in simvastatin-domain-unaffected regions is due to substantial levels of simvastatin integrating into the bilayer and ordering it.

Time-series of BODIPY-PM FLIM images acquired with laser-heated area after 5 µM simvastatin addition

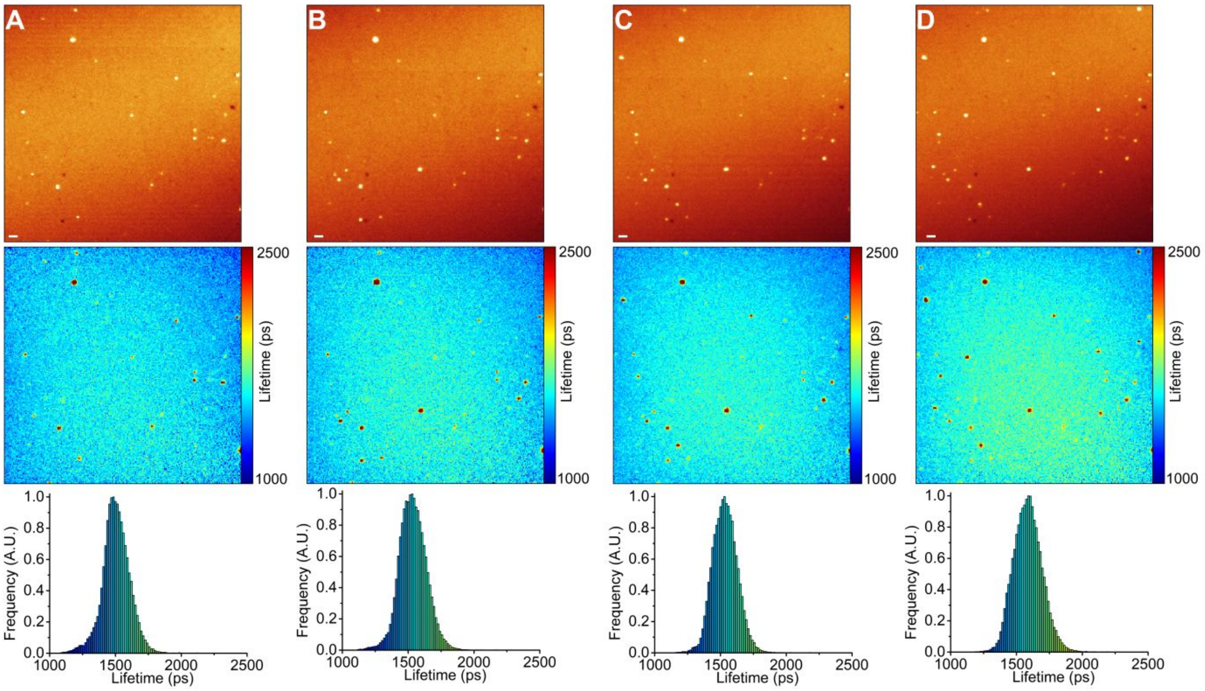

Figure S10. FLIM of BODIPY-PM in DOPC/Chol 60/40 tBLMs. (A) tBLM 3 min simvastatin (5 µM) addition. (B) tBLMs 4 min after simvastatin (5 µM) addition (C) tBLMs 5 min after simvastatin (5 µM) addition. (D) tBLMs 6 min after simvastatin (5 µM) addition. The top panel shows images of fluorescence intensity. FLIM images are shown in the middle panel. The corresponding lifetime histograms are shown in the bottom panel. Scale bars are 1 µm.

To investigate how temperature affects simvastatin-induced phase separation, we increased the laser power and irradiated a small area of DOPC/Chol 60/40 tBLMs following a 5 µM simvastatin addition. The initial and final FLIM measurements of BODIPY-PM were performed at room temperature using standard laser irradiation and are shown in S11. After adding 5 µM of simvastatin and continuously irradiating the sample region, circular and viscous domains were still visible (Fig. S10). Continuous laser-irradiation, which raises the temperature of tBLMs, resulted in the gradual disappearance of certain circular domains (Fig. S10). Importantly, the intensity-weighted fluorescence lifetimes of BODIPY-PM in domain-free regions of the bilayer gradually shifted from about 1500 ps to 1750 ps, especially around the domain-surround areas. This result indicates that upon an increase in temperature, the circular domains can form a single homogeneous phase with the domain-free regions of the lipid bilayer and release simvastatin with cholesterol, which increases the local microviscosity.

Circular domain formation with laser-heated area after 5 µM simvastatin addition

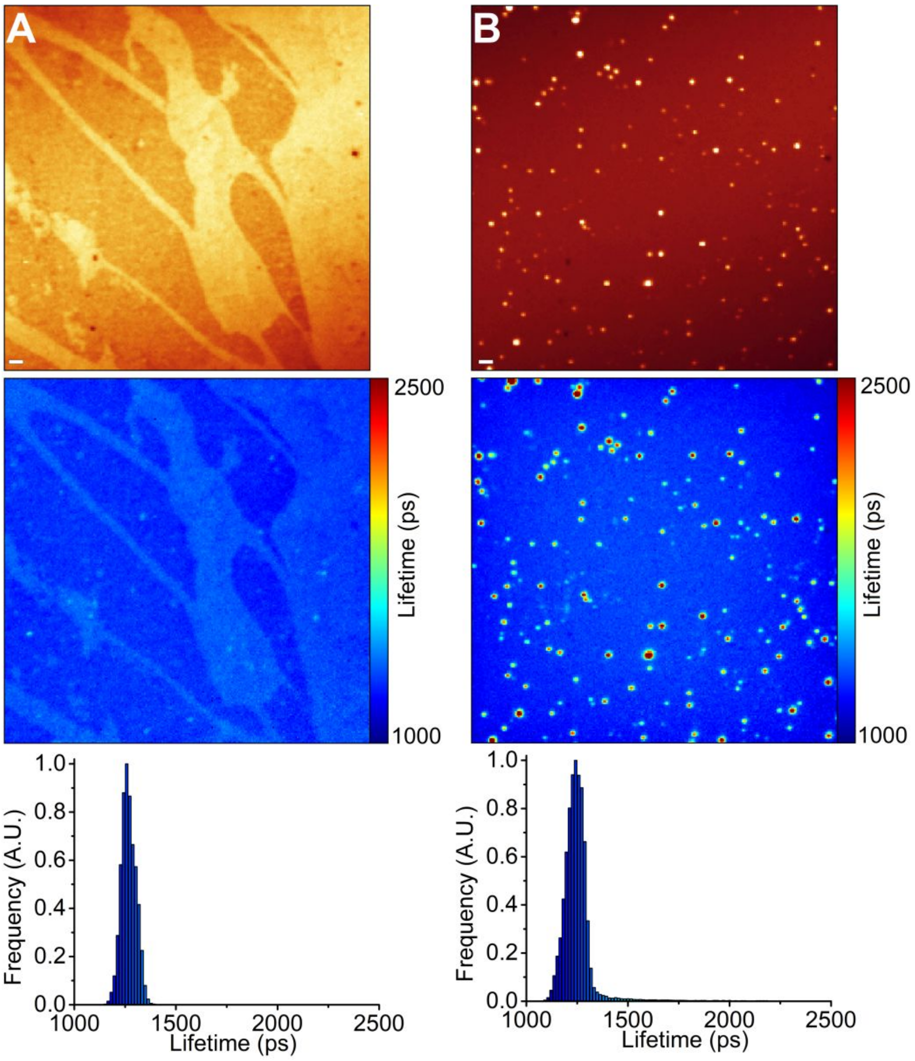

Figure S11. FLIM of BODIPY-PM in DOPC/Chol 60/40 tBLMs. (A) tBLM before simvastatin (5 µM) addition. (B) tBLMs 10 min. after addition of simvastatin (5 µM). FLIM images taken at normal laser irradiation.The top panel shows images of fluorescence intensity. FLIM images are shown in the middle panel. The corresponding lifetime histograms are shown in the bottom panel. Scale bars are 1 µm.

We additionally present the initial and final FLIM measurements of BODIPY-PM in DOPC/Chol 60/40 tBLMs from the region presented in Fig. S10. The initial (Fig. S11A) and final (Fig. S11B) images were obtained with normal laser irradiation and display normal room-temperature microviscosities of tBLMs.

Electrochemical Impedance (EIS) spectra of DOPC and DOPC/Chol 60/40 tBLMs upon exposure to simvastatin and pravastatin

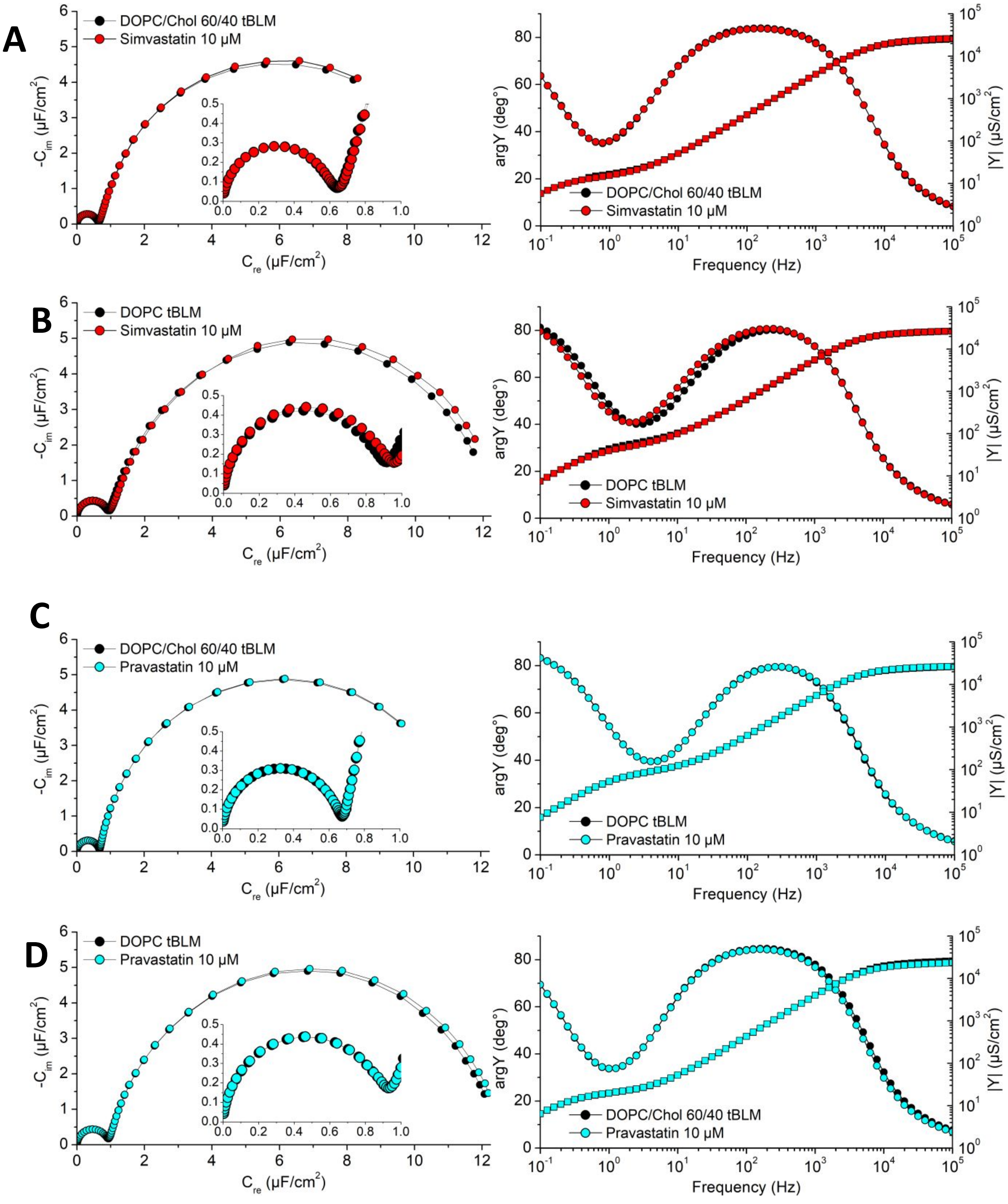

Figure S12. EIS spectra of DOPC and DOPC/Chol 60/40 tBLMs before and 30 min after addition of simvastatin (A, B) or pravastatin (C, D) at concentrations of 10 μM. Showing Cole-Cole complex capacitance plots (left) and Bode phase and admitance modulus plots (right).

We used electrochemical impedance spectroscopy (EIS) to determine if simvastatin-induced phase separation causes any damage to the tBLMs. The addition of simvastatin had no significant effect on

DOPC/Chol 60/40 tBLMs, whereas pure DOPC tBLMs showed a modest shift in phase minima (Fig. S12A and S12B). The slight phase minima shift in DOPC tBLMs following simvastatin addition indicates that the DOPC lipid bilayers become more ordered. In contrast, the addition of pravastatin to either DOPC or DOPC/Chol 60/40 tBLMs produced no observable changes in the EIS spectra. Importantly, we did not observe the formation of any defects or water-filled pores after simvastatin addition, indicating that DOPC lipids in the phase separation boundaries are continuous with simvastatin-induced domains.

FLIM of HEK 293T cells 15 min after simvastatin addition

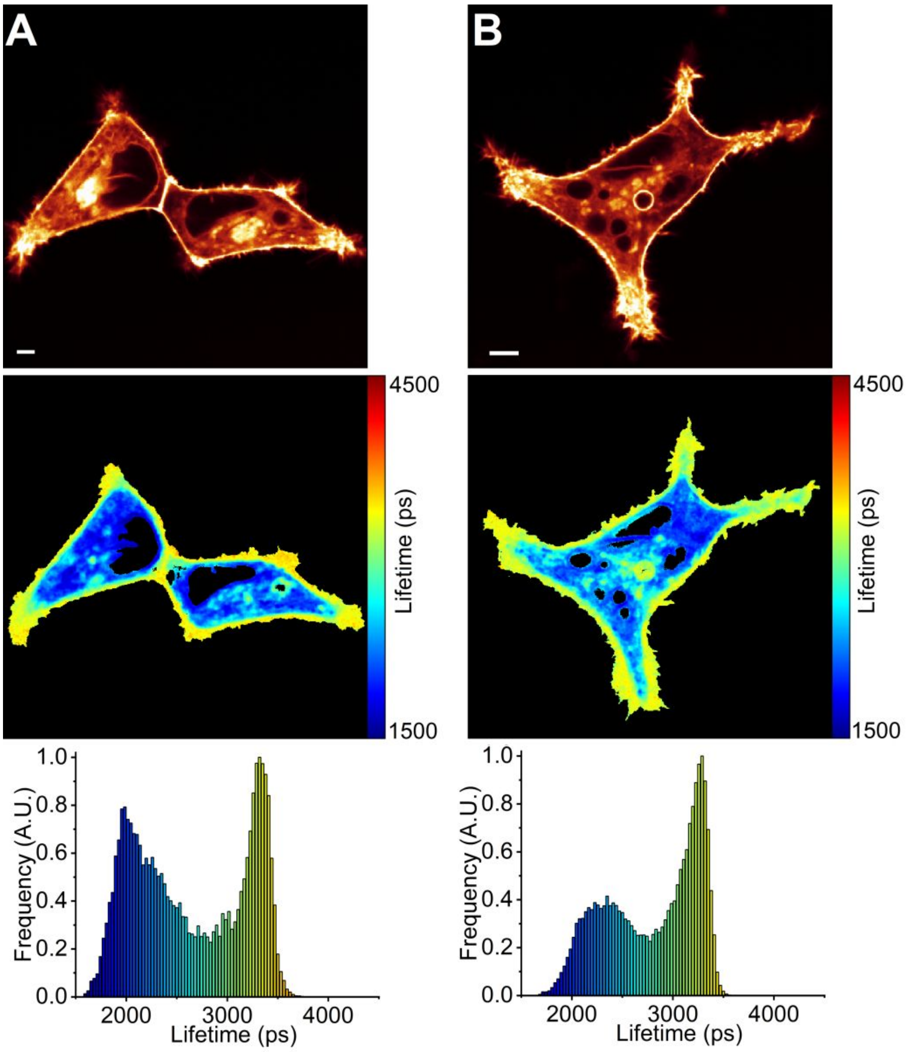

Figure S13. FLIM of BODIPY-PM in live HEK 293T cells. (A, B) HEK 293T cells 15 min after simvastatin addition. The top panel shows images of fluorescence intensity. FLIM images are shown in the middle panel. The corresponding lifetime histograms are shown in the bottom panel. Scale bars are 5 µm.

We decided to incubate the HEK 293T cells with simvastatin for 15 minutes to determine whether the simvastatin-induced morphology changes are transient. After 15 min of simvastatin (10 µM) treatment, cells were washed with PBS, stained with BODIPY-PM in the culture medium, and quickly

imaged afterwards (Fig. S13). We note that the morphology of HEK 293T cells is completely restored after 15 minutes of simvastatin treatment. Additionally, we observed the presence of micron-sized vesicles in the cytoplasm of live cells (Fig. S13).The intensity-weighted fluorescence lifetimes of BODIPY-PM in said vesicles are about 3000–3500 ps, a value closely similar to the plasma membranes of simvastatin-affected cells. We suspect that simvastatin promotes membrane internalization, which leads to the appearance of vesicles inside the cytoplasm.

Imaging of doxorubicin fluorescence intensities in simvastatin or pravastatin-treated A549 cells

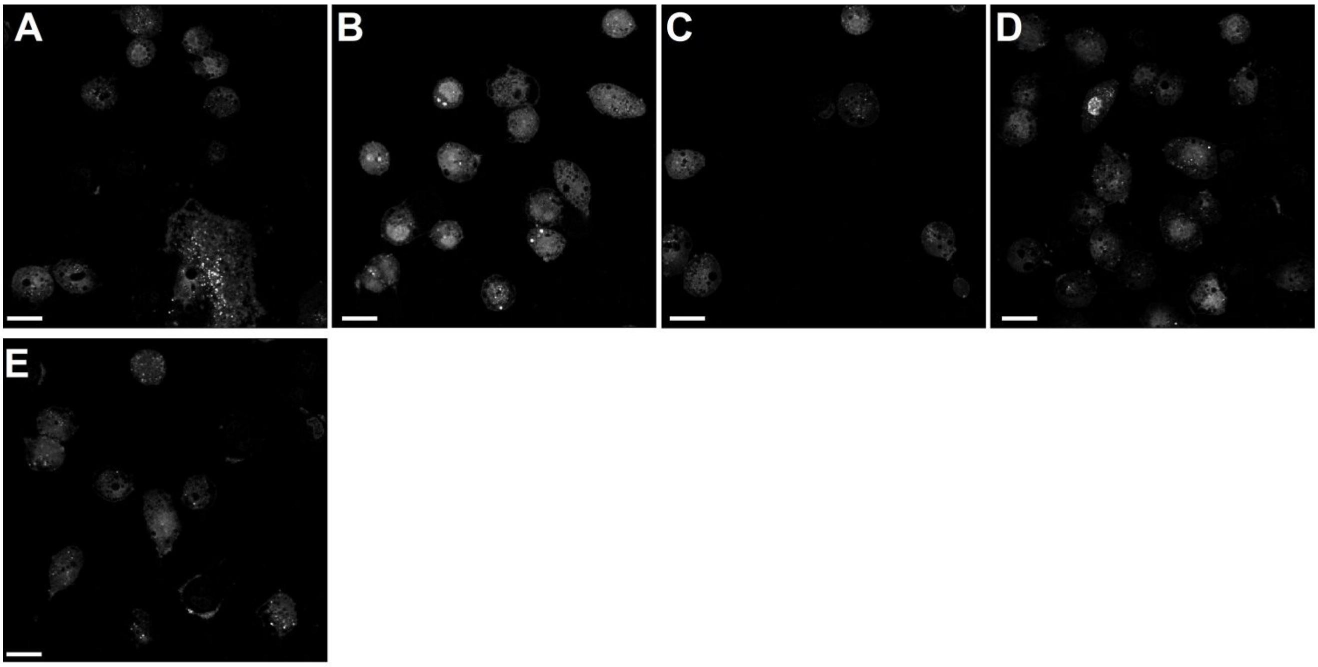

Figure S14. Fluorescence intensity images of doxorubicin in A549 cells. (A) A549 cells stained with doxorubicin following 4 hrs. of simvastatin treatment. (B) A549 cells stained with doxorubicin following 5 min of simvastatin treatment. (C) A549 cells stained with doxorubicin following 4 hrs. of pravastatin treatment. (D) A549 cells stained with doxorubicin following 5 min of pravastatin treatment. (E) A549 cells stained with doxorubicin, cells were not treated with simvastatin or pravastatin. Scale bars are 20 µm.

To study how simvastatin-induced membrane fluidization affects the accumulation of anticancer drugs, we treated A549 cells with statins and measured intracellular drug accumulation. (Fig. S12). To measure the drug accumulation, we chose the fluorescent anticancer drug doxorubicin. In all of the experiments, cells were treated with 10 µM of doxorubicin for 10 min and imaged immediately afterwards. Doxorubicin was excited with a pulsed white light laser line at 480 nm, and fluorescence intensity was measured at 570–650 nm. After 4 hours of simvastatin (Fig. S12A) or pravastatin (Fig. S12C) treatment, A549 cells exhibited similar doxorubicin fluorescence intensities to untreated cells (Fig. S12E). In addition, we did not observe any significant doxorubicin fluorescence intensity differences between control (Fig. S12E) and 5 min of pravastatin treatment (Fig. S12C). Mean doxorubicin fluorescence intensities following different A549 treatments are presented in Fig. S13.We conclude that pravastatin does not fluidize the cellular membranes and does not lead to higher intracellular doxorubicin accumulation. In contrast, A549 cells treated for 5 min with simvastatin

exhibited about twice as high doxorubicin fluorescence intensities compared to the untreated cells, pravastatin-treated cells, or cells that were treated with simvastatin for 4 hrs. (Fig. S12 and S13). Since no significant differences in doxorubicin fluorescence intensities between 4 hrs. of simvastatin treatment and control were observed, we conclude that after 4 hrs, the plasma membranes relax to their initial microviscosities and thus do not lead to increased drug permittivity.

Mean doxorubicin fluorescence intensities in A549 cells with different simvastatin or pravastatin treatments

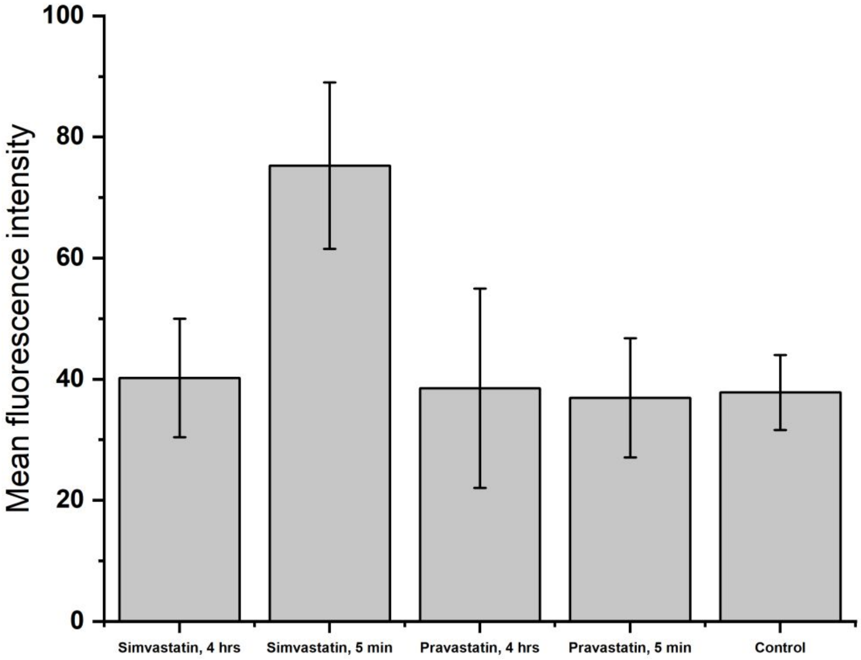

Figure S15. Mean doxorubicin fluorescence intensities with standard deviations in A549 cells following different simvastatin or pravastatin treatments (n=3).
